# Environmental stress promotes the persistence of juvenile traits in olfactory neurons as a protective mechanism

**DOI:** 10.1016/j.isci.2025.113078

**Published:** 2025-07-24

**Authors:** Julien Brechbühl, Ana Catarina Lopes, Dean Wood, Elodie Sauge, Marianne Sidhom, Noah Gilliand, Monique Nenniger Tosato, Frédéric Gachon, Marie-Christine Broillet

**Affiliations:** 1 Department of Biomedical Sciences, Faculty of Biology and Medicine, University of Lausanne, 1011 Lausanne, Switzerland; 2 Institute for Molecular Bioscience, the University of Queensland, St. Lucia, QLD, Australia

**Keywords:** Biological sciences, Animal physiology, Evolutionary developmental biology, Developmental biology

## Abstract

The persistence of juvenile-like properties in specific cell types can provide adaptive advantages to stressful environments. In animals, such traits are well described at the organismal level, but their existence and regulation at the cellular level remain poorly understood. Here, using mice with genetically tagged mature olfactory sensory neurons (OSNs), we show that these neurons can retain immature features in response to environmental stress generated by an increased airflow. This delayed maturation is associated with the sustained nuclear expression of the activating transcription factor 5 (ATF5), which we found to be a potential regulator of this neuronal plasticity. In *Atf5*-deficient mice, this adaptive response fails to occur, leading to the subsequent loss of OSNs under environmental stress. Thus, the identified protective mechanism in the olfactory system may serve to safeguard neuronal integrity and ensure functional adaptation to environmental stress.

## Highlights

• OSNs retain juvenile traits in response to environmental stress
• Environmental stress induces nuclear ATF5 expression in OSNs
• ATF5 deletion phenotypes emerge under environmental stress in OSNs
• Juvenile-like adaptation fails to occur in the absence of ATF5

## Graphical abstract

**Figure:**
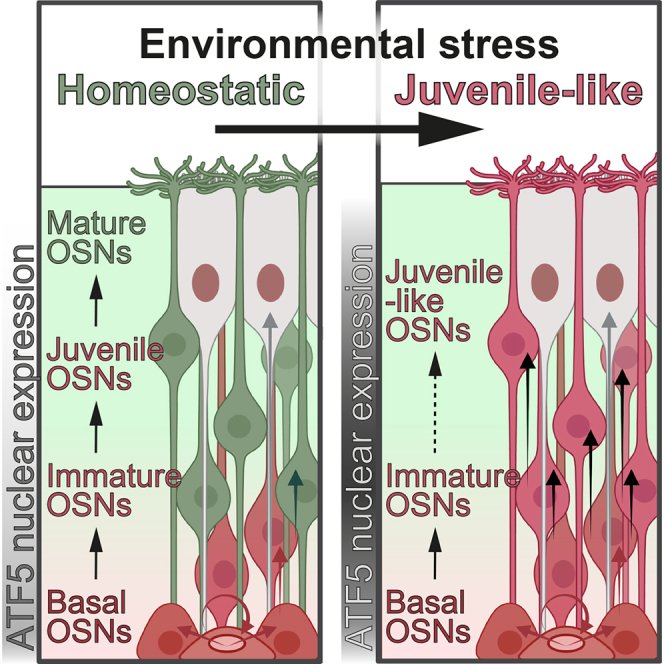

## Introduction

Organisms are constantly exposed to multiple environmental stressors and have developed biological strategies to adapt and prevent cellular injuries. One of such strategies is the modulation of developmental timing, allowing organisms, tissues, or cells to display juvenile-like properties under specific conditions.^1^^,^^2^^,^^3^^,^^4^ These phenomena are observed across multiple species in the animal kingdom and they involve the interplay of genetic and environmental factors that influence maturation processes.^1^^,^^3^^,^^5^^,^^6^ At the species level, neoteny is a well-known example of developmental shift, in which juvenile traits are retained into adulthood, often as an adaptive response to ecological pressures. In the axolotl (*Ambystoma mexicanum*), such evolutionary heterochrony leads to the retention of larval features, including external gills and high regenerative capacity, due to altered endocrine signaling.^6^^,^^7^ At the organismal level, some mammals also exhibit delayed maturation as a strategy for environmental resilience. The naked mole rat (*Heterocephalus glaber*), for example, displays a markedly extended juvenile phase, characterized by delayed aging, cancer resistance, and enhanced longevity, likely reflecting an adaptation to its hypoxic subterranean habitat.^8^^,^^9^ Remarkably, similar mechanisms occur at the tissue and cellular levels, contributing to the maintenance of juvenile-like properties into adulthood under specific environmental conditions. For instance, in the mature mammalian cochlea, an overstimulation signal is required to trigger the differentiation of cells with stem-like potential that remain in the apical turn.^10^ In humans, a delayed maturation is also observed in specific brain regions, helping to preserve the synaptic plasticity essential for learning and memory.^11^ A population of immature neurons, for example, in the amygdala paralaminar nucleus, has been reported to persist into adulthood and to undergo delayed maturation, contributing to olfactory behaviors under environmental influence.^3^ Moreover, cellular states can also revert from a mature to a more juvenile profile in response to regenerative therapies, intrinsic signals, or environmental stimuli. This process, known as rejuvenation, involves active cellular reprogramming mechanisms aimed at restoring youthful functions.^2^^,^^12^ However, this prolonged immaturity may also increase vulnerability to pathologies, including neurodegenerative disorders such as Alzheimer’s disease.^13^ Understanding how environmental inputs shape maturation, and uncovering the cellular mechanisms that regulate these dynamics, is therefore essential.

Given their role as sentinels, olfactory sensory neurons (OSNs) are directly exposed to variations in environmental stimuli.^14^ For example, asymmetrical congestion of the erectile tissues in the right and left nasal cavities generates alternating fluctuations in airflow through the nostrils. While this phenomenon can result from pathological conditions such as nasal obstruction or fixed septal deviation, it is also a normal physiological process known as the natural nasal cycle.^15^^,^^16^ These cycles, regulated by the autonomic nervous system, typically last between 2 and 4 h in humans, with variations across species and individuals. Although less well characterized in rodents, evidence suggests that similar fluctuations in nasal airflow occur and may play a role in modulating odor detection and exposure to environmental agents.^17^ Beyond its role in maintaining nasal humidification and mucociliary clearance, the nasal cycle may also enhance olfactory sensitivity by alternating airflow conditions in each nostril. This dynamic regulation prevents olfactory receptor (OR) saturation and optimizes odorant-receptor interactions, as some molecules bind efficiently under high airflow, while others require prolonged contact time in low-flow conditions.^15^^,^^17^ Moreover, airflow, which varies significantly depending on environmental conditions,^14^ also serves as a vector for environmental insults, such as airborne pollutants, viruses, or volatile chemicals. Altogether, these heightened environmental stimulations cause cellular damage and lead to the accumulation of misfolded proteins in the endoplasmic reticulum (ER) of OSNs.

To cope with this cellular stress, OSNs use their intrinsic plasticity and activate pro-survival mechanisms, such as the unfolded protein response (UPR).^18^ The UPR is a conserved cellular stress response that restores ER homeostasis by transiently reducing global protein translation, promoting the degradation of misfolded proteins, and upregulating molecular chaperones to enhance proper protein folding. This adaptive mechanism helps OSNs maintain their function under challenging environmental conditions.^19^^,^^20^ Specifically, the activating transcription factor 5 (ATF5), which belongs to the ATF/3′,5′-cyclic adenosine monophosphate (ATF/cAMP) response element-binding (CREB) family, a large group of basic region leucine zipper (bZIP) proteins involved in diverse transcriptional regulatory functions,^21^ is a key regulator of the UPR process that plays an essential role in OSN homeostasis.^22^ Under increased protein-folding demand, ATF5 expression is indeed upregulated at the post-transcriptional level and through enhanced nuclear translation in immature neurons,^23^^,^^24^^,^^25^^,^^26^ supporting their development and ensuring proper OR gene selection as well as their terminal differentiation and survival into mature OSNs.^22^^,^^25^^,^^27^^,^^28^^,^^29^^,^^30^ Furthermore, *Atf5* genetic inactivation in mouse is associated with significant apoptosis in OSNs that results in impaired odorant detection or anosmia at birth, severely compromising the ability of pups to locate their mother’s nipples and leading to perinatal viability defects.^22^^,^^27^^,^^31^ Nevertheless, it remains to be determined whether OSNs also use mechanisms that maintain juvenile-like cellular properties as part of their adaptive response.

**Figure 1:**
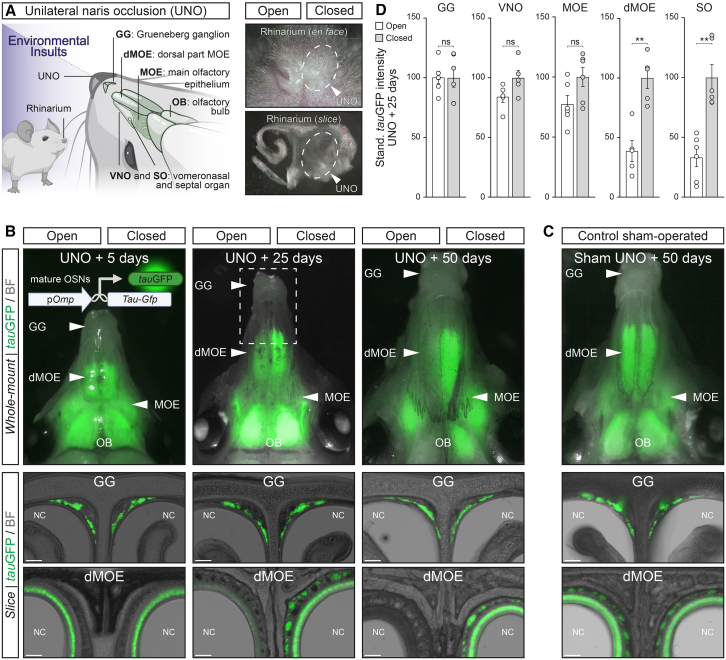
Increased environmental stimulations modulate the *tau*GFP signal in the mouse olfactory system (A) Schematic representation of a mouse head and its olfactory subsystems (left; created with BioRender.com), the Grueneberg ganglion (GG), the main olfactory epithelium (MOE) and its dorsal part (dMOE), the vomeronasal organ (VNO), the septal organ of Masera (SO), the olfactory bulb (OB) as well as the localization of the rhinarium, where the unilateral naris occlusion (UNO) procedure is performed. The inspection of the UNO efficiency is observed here in *en face* and *slice view* (right) of a heterozygous OMP-GFP mouse after 25 days of UNO. The UNO procedure allows the simultaneous establishment of a stimulated (open) and protected side of the nose (closed; highlighted here with the dashed white lines). (B) The genetic strategy used to observe the maturity of olfactory sensory neurons (OSNs) is indicated (top left). The promoter of the olfactory marker protein (p*Omp*) is used to control the persistent expression of the *tau*GFP (in green). Whole-mount and coronal slice observations of the different *tau*GFP signal intensities after UNO. Olfactory subsystems are differentially impacted by environmental stimulations across days post-surgical procedure (UNO +5/25/50 days). The region in the dashed white rectangle (middle), where the GG and the dMOE are observed in whole-mount view (highlighted in the Figure S1A). (C) Representative control sham-operated mouse after 50 days post-procedure (sham UNO + 50 days). (D) Quantification of the level of the *tau*GFP signals is performed and compared between open (in white) and closed (in gray) sides in the different olfactory subsystems, 25 days post-UNO. Scale bars represent 100 μm in (B) and (C). For the morphological aspect, nasal cavities (NC) are indicated in (B) and (C). Bright-field illumination (BF, in B and C). Data are expressed as mean ± SEM with aligned dot plots for ≥5 mice in (D). For comparisons between conditions, two-tailed Student’s t test or Mann-Whitney U test are used, ∗∗*p* < 0.01, ns for non-significant in (D).

In the mouse nose, olfactory neurons are localized in different olfactory subsystems that display distinctive morphological organization and physiological characteristics.^32^ In the main olfactory epithelium (MOE; Figure 1A), OSNs are organized within a regenerating pseudostratified neuroepithelium that displays a characteristic layered structure. This epithelium comprises basal progenitor cells, including horizontal basal cells (HBCs) and globose basal cells (GBCs), which act respectively as long- and short-term reservoirs for both neuronal and non-neuronal lineages.^33^ These basal cells then differentiate into immature, juvenile, and finally mature OSNs. Once the differentiation is complete, mature OSNs express the olfactory marker protein (OMP), a hallmark of neuronal maturity across the entire olfactory system.^34^ They become fully functional by expressing their specific OR and the sensory transduction proteins, including the adenylyl cyclase type III (ACIII) and the cyclic nucleotide-gated channel alpha 2 (CNGA2), at the level of their neuronal cilia in direct contact with the mucus of the nasal cavity.^35^^,^^36^ Moreover, environmental accessibility is strongly enhanced in the dorsal part of the MOE (dMOE; Figure 1A), where the primary nasal airflow occurs during inhalation.^17^^,^^33^^,^^37^ In contrast, in the Grueneberg ganglion (GG; Figure 1A), glial cells wrap the ciliated sensory neurons, which express non-canonical signaling elements such as the particulate guanylyl cyclase G (GC-G).^38^ The GG neurons are clustered within a grape-shaped structure, which is protected from the nasal cavity by a permeable keratinized epithelium.^39^ This morphological feature allows both the temperature-dependent detection of volatile chemicals giving danger information, such as the alarm pheromones and the predator-derived kairomones, and a protection from the environment.^39^^,^^40^^,^^41^^,^^42^ Interestingly, the regenerative ability of the different olfactory neurons also differs across the olfactory subsystems. Indeed, in the MOE, as well as in the septal organ of Masera (SO; Figure 1A) and the vomeronasal organ (VNO; Figure 1A), pools of basal cells enable a constant neuronal turnover, facilitating the tissue regeneration of both olfactory neurons and supporting cells with a mean neuronal half-life of approximately 25 days in the MOE.^32^^,^^36^^,^^43^ In contrast, in the GG, neuronal plasticity appears to be restricted to perinatal stages, as this olfactory subsystem seems to be fully developed and functional at birth.^39^^,^^44^

The impact of external factors on olfactory neurons has been studied using the unilateral naris occlusion (UNO) method^45^ (Figure 1A), which involves physically sealing one nostril of rodents to mimic the natural nasal cycles^15^ in a controlled manner (efficiency and duration). This procedure offers significant advantages as the “open” side is naturally exposed to increased environmental stimulation without introducing surgery-related artifacts or alterations in cellular turnover,^46^^,^^47^ while the “closed” side serves as an internal reference for the experiments.^14^ On the short term, this technique has been used in previous studies to demonstrate the reversible activity-dependence of various transduction genes in mature OSNs of the MOE.^48^^,^^49^ It has also been used to show that olfactotoxic and chemical stressors lead to the translation of UPR members, independently of odorant stimulation.^19^ Interestingly, the expression of OMP itself was found to be influenced by UNO, at least in the MOE, a phenomenon attributed to a potential sensory adaptation.^50^ While the induced impact of chemical stress on the olfactory system was observed, the neuronal survival mechanism linking environmental stress, the protective UPR system, and the OSNs fate was missing.

Here, we took advantage of a mouse line with genetically tagged mature olfactory neurons to challenge the OSNs response to persistent heightened airflow input. We observed that this environmental stress delays OSN maturation, a phenomenon that is positively correlated with environmental accessibility as well as with the nuclear expression of ATF5. We next genetically disrupted ATF5 expression and further demonstrated that the occurrence of juvenile-like neurons was prevented. Our findings reveal, at the cellular level, a unique olfactory protective mechanism where ATF5 may act as a molecular determinant for the adaptive persistence of juvenile traits in OSNs observed under enhanced environmental stimulations.

## Results

### Enhanced environmental stimulation promotes the persistence of juvenile traits in olfactory sensory neurons

We took advantage of the UNO method (Figure 1A) to examine the effects of enhanced environmental stimulations on the mouse olfactory system (Figures 1B and S1). We used the OMP-green fluorescent protein (OMP-GFP) mouse model where all mature OSNs express GFP fused with the tubulin-associated unit (TAU).^51^ These mice were initially developed to observe a global cellular distribution of GFP and to allow OSNs axonal tracing to the first integrative part of the brain, the olfactory bulb^52^ (OB; Figure 1A). Remarkably, this genetic association between TAU and GFP (mentioned as the *tau*GFP signal) permanently tags^53^^,^^54^^,^^55^ newly matured OSNs. Furthermore, the continuous observation of this *tau*GFP signal also allowed us to follow their neuronal fate.

In whole-mount upper views of OMP-GFP mouse head preparations (*whole-mount*; Figure 1B) performed on different days post UNO procedure (P0), we first observed a time-dependent downregulation of the *tau*GFP signal (Figure 1B) in the dMOE on the open naris side, where the environmental stimulation is increased (open; Figure 1B). Compared with the closed or protected naris side (closed; Figure 1B) or with the control sham-operated mice (Figure 1C), this visual decrease in *tau*GFP intensity was relevant 25 days after the UNO procedure (UNO + 25 days; Figure 1B) and it was particularly marked after 50 days (UNO + 50 days; Figure 1B) in the dMOE (Figures 1A and 1B). To refine this observation, we next looked at slice preparations of the different olfactory subsystems and we indeed noticed a significant discrepancy in their *tau*GFP signal (*slice*; Figures 1B and S1), which we found to be remarkably linked with their anatomical accessibility to environmental stimulations. Indeed, no significant ipsilateral time-dependent impact of environmental stimulations was observed in the GG, which neurons are naturally protected from the environment, 25 days post-procedure (Figures 1B, 1D, S1A, and S1B; closed vs. open side; *N*_mouse_ = 6, +0.2% *tau*GFP intensity, ns) or in the VNO, which is also connected to external stimuli via a specific duct and thus less sensitive to the UNO procedure^56^ (Figures 1D and S1C). While the effect observed on the *tau*GFP signal was moderate in the MOE, where the airflow is naturally dampened by the morphology of the turbinates,^37^ it was clearly initiated in the dMOE and progressively extended along the caudal MOE (Figures 1A, 1B, 1D, S1A, and S1B; closed vs. open side; *N*_mouse_ = 5, −61.2% *tau*GFP intensity, ∗∗). In contrast, the influence of environmental stimulation was robust in the SO subsystem (Figures 1D and S1C), whose neurons are fully exposed to chemical cues. Remarkably, the reversibility of this phenomenon proved to be rapid, as approximately five days post naris reopening, the *tau*GFP signal was completely restored (Figures S1D–S1F), strongly suggesting that this phenomenon is highly involved as a defensive mechanism against an unfavorable environment. Moreover, confirming the importance of this phenomenon throughout the animal’s life, the sensitivity to this external stimulation is independent of the animal’s age, as demonstrated when the UNO procedure was performed on adult animals (postnatal month [PM] 2–14) (Figure S1B). We selected these two olfactory regions (dMOE and GG) for comparative experiments of environmental exposure.

We thus further studied, at the cellular level, the phenomenon observed in the dMOE, 25 days after the increased environmental exposure. In this temporal window, corresponding to the mean half-life of the OSNs, we first verified the absence of imbalance in the cellular renewal dynamics under environmental stimulations. We found no significant difference in the general OSNs density in the dMOE (closed side: 1.49 ± 0.12 × 10^−2^ nuclei/μm^2^; open side: 1.50 ± 0.06 × 10^−2^ nuclei/μm^2^; *N*_mouse_ = 5; ns) as well as no significant variation in the proliferation rates (BrdU labeling; Figures S2A and S2B) and no significant difference in apoptosis thanks to the level of cleaved caspase-3^57^ (CASP3 labeling; Figures S2C and S2D) between the epithelia of the open (exposed) and closed (protected) sides or with sham-operated mice (sham; Figures S2B and S2D). The cellular density as well as the ability of the dMOE to continuously regenerate is thus conserved in spite of the increase in environmental stimulations. Thus, the previously observed decrease in *tau*GFP signal at the level of the OE (Figure 1B) is therefore not due to a reduction in the number of OSNs composing the dMOE, but it rather strongly suggests that, during these 25 days, the newly generated OSNs, corresponding to approximatively half of the total OSNs composing the epithelium, remain in an intriguing *tau*GFP negative or immature-like state.

**Figure 2:**
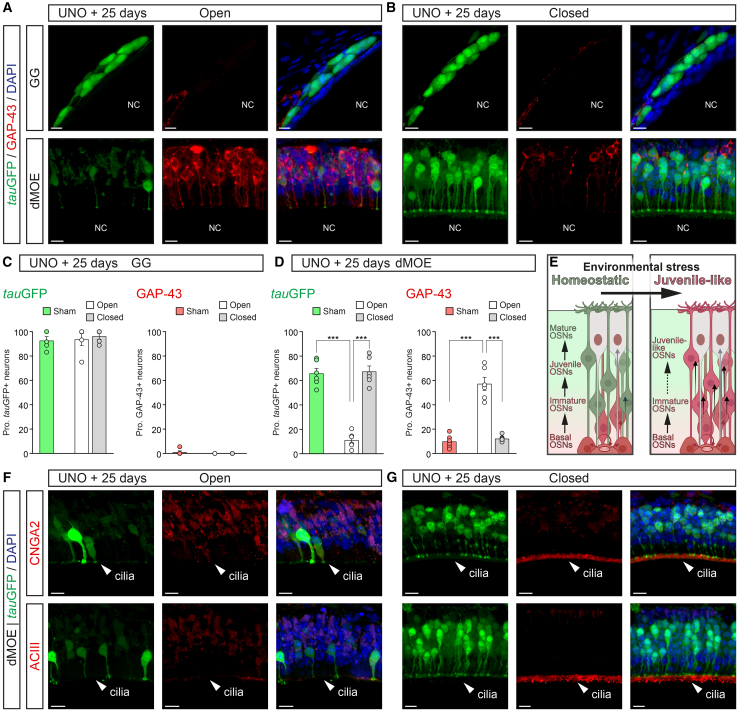
Increased environmental stimulations induce the presence of juvenile-like neurons in the dMOE (A and B) Immunohistochemistry for the marker of immature neurons, the growth-associated protein 43 (GAP-43, in red), on heterozygous OMP-GFP mice, after 25 days of UNO. Representative immunostainings from the GG and from the dMOE are shown for the open (A) and the closed (B) side. The *tau*GFP signal (left, in green), the GAP-43 (middle), and the merge views (right) are shown. (C and D) Thanks to the maturity-dependent *tau*GFP signal (in green), the quantification of the level of *tau*GFP+ and GAP-43+ neurons is performed and compared between sham-operated (sham), open (in white), and closed (in gray) sides both in the GG (C) and in the dMOE (D). (E) A schematic summary of the observed maturity-dependent phenomenon that takes place in the dMOE after an increase of environmental stress (created with BioRender.com). The homeostatic state of the epithelium is replaced by a juvenile state. Basal, immature, juvenile, mature, and juvenile-like OSNs are indicated. Black arrows indicate the direction of neuronal development. (F and G) Representative immunohistochemistry of the cyclic nucleotide gated channel subunit alpha 2 (CNGA2, in red) and of the adenylyl cyclase III (ACIII, in red) in the open (F) and closed (G) side of the dMOE from a heterozygous OMP-GFP mice, after 25 days of UNO. The cilia region (white arrowhead) is indicated to highlight the conventional localization of ACIII/CNGA2. For the morphological aspect, nasal cavities (NC) are indicated in (A) and (B). Nuclei are counterstained with DAPI (DAPI, in blue) in (A), (B), (F), and (G) and is used to quantify the proportion (Prop.) of *tau*GFP+ and GAP-43+ neurons according to the number of DAPI+ nuclei in (C) and (D). Scale bars represent 10 μm in (A), (B), (F), and (G). Data are expressed as mean ± SEM with aligned dot plots for ≥5 mice in (C) and (D). For comparisons between conditions, two-tailed Student’s t test or Mann-Whitney U test are used, ∗∗∗*p* < 0.001 in (C) and (D).

Confirming our hypothesis, we observed that environmental stimulation led to the presence of juvenile-like neurons. Specifically, we observed about 75% reduction in the number of *tau*GFP+ OSNs (Figures 2A–2D), which may result from the combined effect of the natural turnover occurring over this 25-day period (leading to the loss of about half of the pre-existing mature neurons) and a potential delay in the maturation of newly generated OSNs (further lowering the proportion of the mature neuronal population). In addition, more than 65% of the neurons expressed the growth-associated protein 43 (GAP-43; Figures 2A–2D), a well-established marker of neuronal immaturity,^58^ compared to the approximately 10% typically observed under baseline conditions. Moreover, the stimulated dMOE displayed an overall increase in the expression of the neuron-specific class III β-tubulin (Tuj1; Figures S3A and S3B), a marker of early neuronal differentiation, predominantly enriched in juvenile OSNs and weakly expressed in mature OSNs.^22^^,^^59^ We ruled out the occurrence of a rejuvenation process under this increased environmental exposure since these GAP-43/Tuj1-positive OSNs did not co-express the persistent *tau*GFP signal (Figures 2A and S3A). This phenomenon was not observed in the GG (Figure 2A). Thus, both the conserved neuronal turnover (Figure S2) and the immature neuronal state observed on the open side, which is stimulated by the environment compared to a homeostatic state, can now be explained by the retention of juvenile-like features in the dMOE epithelium (homeostatic vs. juvenile-like; Figure 2E). Accordingly, the remaining expression of the CNGA2 and of the ACIII in the olfactory neurons of the open side is mostly intracellular and not found in the neuronal cilia (Figures 2F, 2G, S4A, and S4B), where these key signaling elements are usually found in functional mature olfactory neurons.

Thanks to our genetic approach using *tau*GFP labeled OSNs, we here show that, in the presence of increased environmental stimulations, most OSNs display juvenile-like traits, as the expression of their typical neuronal markers of immaturity/maturity and the ciliary targeting of their signaling proteins were significantly altered.

### Enhanced environmental stimulation drive the olfactory nuclear expression of ATF5

**Figure 3:**
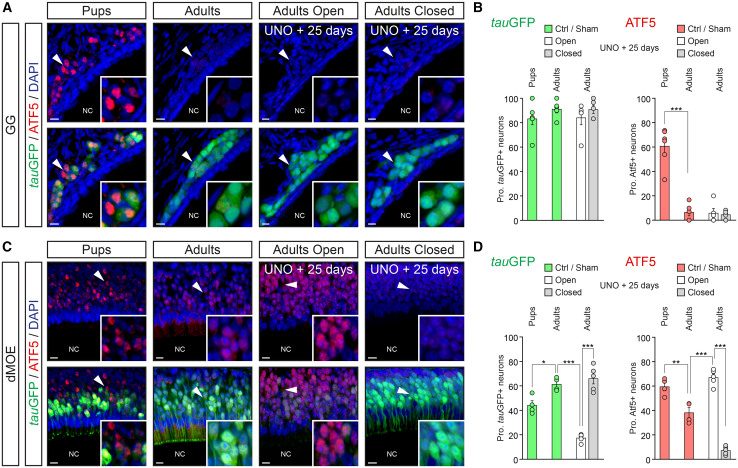
The olfactory nuclear expression of ATF5 is correlated with the increased exposure to environmental stimulations (A and C) Immunohistochemistry for the activating transcription factor 5 (ATF5, in red) on heterozygous OMP-GFP mice, performed on perinatal (pups) or adult (adults) stages of life, as well as after 25 days of UNO (adults open/adults closed). Representative immunohistochemistry for the GG (A) and for the MOE (C). (B and D) Quantification of the proportion of *tau*GFP+ and ATF5+ neurons for control non-operate pups or adult sham-operated (Ctrl/sham), adult open (in white) or closed (in gray) sides both in the GG (B) and in the dMOE (D). White arrowheads indicate the nuclear zoom in view in (A) and (C). For the morphological aspect, nasal cavities (NC) are indicated in (A) and (C). Nuclei are counterstained with dapi (DAPI, in blue) in (A) and (C) and is used to quantify the proportion (Prop.) of *tau*GFP+ and ATF5+ neurons according to the number of DAPI+ nuclei in (B) and (D). Scale bars represent 10 μm in (A) and (C). Data are expressed as mean ± SEM with aligned dot plots for ≥4 mice in (B) and (D). For comparisons between conditions, two-tailed Student’s t test or Mann-Whitney U test are used, ∗*p* < 0.05, ∗∗*p* < 0.01, ∗∗∗*p* < 0.001 in (B) and (D).

We searched for a molecular sensor of increased environmental stimulations in these mice OSNs, and we focused on a UPR member, ATF5, whose expression is regulated by ER stress at the post-transcriptional level and its genetic deficiency has been shown to profoundly affect immature neuron production.^23^^,^^26^ We first verified if the nuclear expression of ATF5 was differentially affected by environmental stimulations in the GG and in the dMOE. We started our experiments at perinatal stages (pups; Figure 3), as birth represents a unique conversion from a protected to a stimulated environment, known to naturally challenge OSNs to environmental stress and active neurogenesis.^60^^,^^61^^,^^62^ We observed, in OMP-GFP newly born pups (P0), a nuclear expression of ATF5 in both GG (Figures 3A and 3B) and dMOE neurons (Figures 3C and 3D), suggesting the activation, at birth, of a UPR-related process in these two olfactory subsystems. We next performed similar assessments in adult mice (PM 3–5) (adults; Figure 3), where natural interactions with environmental stimulations are reduced,^63^ especially in the fully mature GG, that is covered by a protective keratinized epithelium. We found that the proportion of ATF5 nuclear expression was indeed significantly downregulated in both subsystems (Figures 3B and 3D). Remarkably, the GG was particularly affected, as a decrease of almost 90% of ATF5+ neurons was observed (Figure 3B) while it was around 30% in the dMOE (Figure 3D). We finally verified the nuclear expression of ATF5 after 25 days of the UNO procedure in these adult mice (PM 3–5) (adults open/closed; Figures 3B and 3D) and observed no variation in the GG (Figures 3A and 3B), while a significant upregulation occurred in the exposed open side of the dMOE (Figures 3C and 3D).

In summary, we observed a constitutive dMOE adaptation to environmental stimulations that is correlated with the nuclear expression level of ATF5.

### The absence of ATF5 expression impaired the persistence of juvenile traits in olfactory sensory neurons

**Figure 4:**
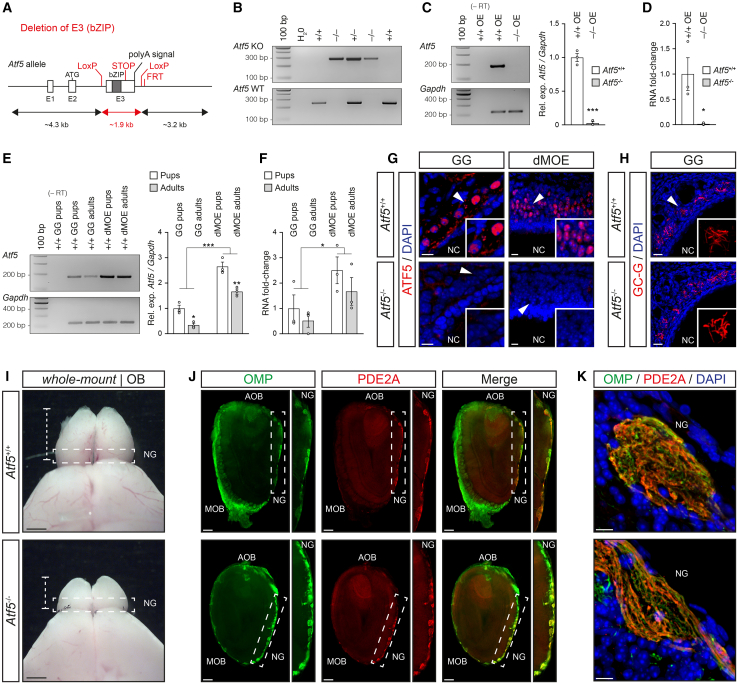
The genetic invalidation of *Atf5* affects differently the olfactory neural circuitries (A) Schematic representation of the Cre-LoxP-based strategy used for the deletion of the exon 3 containing bZip domain for the generation of *Atf5* null mice. (B) Representative genotyping of wild-type (WT) and knockout (KO) alleles of different *Atf5* littermates (+/+; +/−; −/−). H_2_O is used as negative control of DNA amplification. (C) Gene expression profile of the *Atf5* transcripts in mouse OE (mix of MOE, dMOE, and SO epithelia) from *Atf5*^+/+^ (+/+ OE) and *Atf5*^−/−^ (−/− OE) performed by RT-PCR (left) and its semi-quantification (right). (D) Gene expression profile of the *Atf5* transcripts in mouse OE from *Atf5*^+/+^ (+/+ OE) and *Atf5*^−/−^ (−/− OE) performed by RT-qPCR. (E) Gene expression profile of the *Atf5* transcripts found in the GG and dMOE from pups and adult *Atf5*^+/+^ mice performed by RT-PCR (left) and its semi-quantification (right). (F) Gene expression profile of the *Atf5* transcripts found in the GG and dMOE from pups and adult mice performed by RT-qPCR. Gapdh is used as a reporter gene in (C) through (F), and negative controls are performed by omitting the reverse transcription phase (-RT) in (C) and (E). Ladder of 100 base pairs (bp) in (B), (C), and (E). (G) Immunohistochemistry for ATF5 (in red; here from Abcam) shows the absence of ATF5 nuclear signal in *Atf5* null pups both in the GG and dMOE. (H) Anti-pGCG (GC-G, in red) immunostaining shows the expression of the GC-G signaling protein in the GG of both genotypes. (I) Whole-mount view of the OB from *Atf5*^+/+^ and *Atf5*^−/−^ adult mice. White dashed lines show a representative localization of OB width, and dashed white rectangles indicate NG localization. (J) The double immunostaining performed with anti-OMP (OMP, in green) and anti-PDE2A (PDE2A, in red) on OB slices shows the presence of the GG-related NG in both genotypes. Dashed white rectangles indicate NG regions that are zoomed-in (right). For the morphological aspect, the main olfactory bulb (MOB), the accessory olfactory bulb (AOB), and the necklace glomeruli region (NG) are indicated. (K) High-power views of one specific OMP/PDE2A + NG glomerulus per genotype. Nuclei are counterstained with DAPI (DAPI, in blue) in (G), (H), and (K). White arrowheads indicate zoom-in view regions in (G) and (H). Scale bars represent 10 μm in (G) and (K), 20 μm in (H), and 250 μm in (I) and (J). Data are expressed as the relative expression between Atf5 and Gapdh (Rel. exp Atf5/Gapdh) in (C) and (E) or as an RNA fold change in (D) and (F) and represented as mean ± SEM with aligned dot plots for ≥3 mice in (C) through (F). For comparisons between genotypes and conditions, two-tailed Student’s t test or Mann-Whitney U test are used, ∗*p* < 0.05, ∗∗∗*p* < 0.001.

We then postulated that an *Atf5* deficiency would prevent the retention of juvenile traits that takes place in the dMOE under enhanced environmental stimulations. We thus generated a knockout mouse model in which the deletion of the bZIP region was performed, fully invalidating ATF5 function (Figure 4A). The genetically different littermates were then obtained by crossing heterozygous mice (Figure 4B). As previously reported for analogous *Atf5* knockout model, only around 5% (*N*_*Atf5*_^−/−^/*N*_total_ = 18/386) of the total adult mice were genotyped as null mice due to a severe perinatal viability defect. To further validate our mouse model, we next compared the relative expression of the *Atf5* gene in the olfactory epithelium (OE; mix of MOE, dMOE, and SO epithelia) from *Atf5*^+/+^ and *Atf5*^−/−^ adult mice (PM 4–9) (Figures 4C and 4D). RT-PCR (Figure 4C) and RT-qPCR (Figure 4D) analysis demonstrated the absence of *Atf5* transcript in *Atf5*^−/−^ mice. Then, comparing *Atf5* gene expression in the GG and in the dMOE by RT-PCR (Figure 4E) and RT-qPCR (Figure 4F), we also noticed that its expression was proportionally higher in the dMOE. Moreover, and compared to newborn mice (P0–6), we found that this *Atf5* expression was downregulated in adult mice, both in the GG and in the dMOE (Figures 4E and 4F). As expected, we next found, at the protein level, a nuclear expression of ATF5 in *Atf5*^+/+^ pups (P0), both in the GG and in the dMOE, while it was totally absent in *Atf5*^−/−^ pups (P0) (Figure 4G). Together, these observations thus not only substantiate our previous profile of ATF5 protein expression (Figures 3A and 3C) but also confirm an *Atf5*-related regulation process acting both at the transcriptional and translational levels that might be triggered by environmental stimulations. Unless otherwise specified, *Atf5*^+/+^ and *Atf5*^−/−^ littermates were therefore used for further experiments.

We first looked at the functional impact of the *Atf5* genetic deletion on the GG circuitry, and observed that its key signaling element, the particulate guanylyl cyclase G (GC-G; Figure 4H) was still expressed in the GG cilia. We then observed a profound size alteration of the olfactory bulb due to this genetic deletion (Figure 4I; *Atf5*^+/+^: *N*_mouse_ = 4, 3.4 ± 0.0 mm; *Atf5*^−/−^: *N*_mouse_ = 4, 2.1 ± 0.1 mm; ∗∗∗), a phenotype previously linked to the lack of glomerular arrangements in the main olfactory bulb (MOB; Figure 4J) and in the accessory olfactory bulb (AOB; Figure 4J), respectively emerging from the OE (including the MOE, dMOE and the SO) and VNO connections.^22^^,^^64^ Interestingly, we found that the axonal projections of the GG into their OMP-phosphodiesterase 2A (PDE2A) positive structure,^65^ the so-called necklace glomeruli complex (NG; Figure 4J), were still present (Figures 4J and 4K), not only confirming that the GG circuitry exist in *Atf5*^−/−^ mice (at least from GG neurons to NG) but this also strengthens the notion that the absence of a functional ATF5 protein differentially impacts the mouse olfactory subsystems.

**Figure 5:**
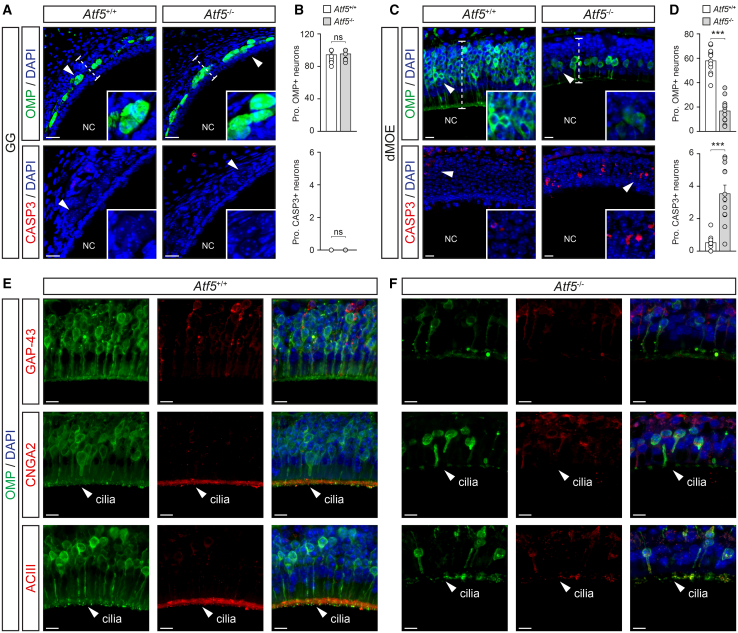
Neuronal maturity and apoptosis of the GG and of the dMOE are differently impacted by the *Atf5* genetic invalidation (A–D) Representative immunohistochemistry for the OMP (in green) and for the CASP3 (in red) respectively used to investigate the maturity and apoptotic levels in the GG in (A) and (B) and in the dMOE in (C) and (D) of *Atf5*^+/+^ (in white) and *Atf5*^−/−^ (in gray) mice. (B and D) The precise quantifications of the proportion of OMP+ or CASP3+ neurons for the GG (B) and for the dMOE (D) are shown. (E and F) Double immunohistochemistry for the OMP (in green) and for the GAP-43 or CNGA2 or ACIII (all in red) are illustrated for *Atf5*^+/+^ (E) and *Atf5*^−/−^ (F) mice. The cilia region (white arrowhead) is indicated to highlight the normal localization of CNGA2/ACIII in (E) and (F). White dashed lines designated a representative localization of epithelium width. For the morphological aspect, nasal cavities (NC) are indicated in (A) and (C). Nuclei are counterstained with DAPI (DAPI, in blue) in (A), (C), (E), and (F) and are used to quantify the proportion (Prop.) of OMP+ and GASP3+ neurons according to the number of DAPI+ nuclei in (B) and (D). Scale bars represent 20 μm in (A), and 10 μm in (C), (E), and (F). Data are expressed as mean ± SEM with aligned dot plots for ≥6 slices emerging from at least 3 different mice per genotype in (B) and (D). For comparisons between genotypes, two-tailed Student’s t test or Mann-Whitney U test are used, ∗∗∗*p* < 0.001, ns for non-significant in (B) and (D).

Using immunohistochemistry in adult mice (PM 3–7), we further characterized the impact of the *Atf5*-genetic deletion on the neuronal maturity and apoptosis in the GG and in the dMOE (Figures 5A–5D). Based on the OMP staining, profound alterations were indeed observed in the dMOE (Figures 5C and 5D) with a reduction of approximately 70% in OMP+ neurons (Figures 5C and 5D; *Atf5*^+/+^: *N*_mouse_ = 5, *n*_slice_ = 12, 58.2 ± 3.0%; *Atf5*^−/−^: *N*_mouse_ = 3, *n*_slice_ = 13, 16.9 ± 2.7%; ∗∗∗). Moreover, measuring nuclear pyknosis (Figure 5C; *Atf5*^+/+^: *N*_mouse_ = 3, *n*_slice_ = 10, 0.3 ± 0.1%; *Atf5*^−/−^: *N*_mouse_ = 3, *n*_slice_ = 12, 2.6 ± 0.2%; ∗∗∗) and CASP3 levels (Figures 5C and 5D; *Atf5*^+/+^: *N*_mouse_ = 3, *n*_slice_ = 10, 0.5 ± 0.2%; *Atf5*^−/−^: *N*_mouse_ = 3, *n*_slice_ = 12, 3.6 ± 0.2%; ∗∗∗), both markers of apoptosis,^57^ revealed a significant increase in apoptotic cells, by approximately 85%, in the dMOE. This reinforced our observations and confirmed previous reports performed on the MOE.^22^^,^^27^ Moreover, we observed an overall reduction of approximately 30% in the width of the dMOE epithelium, a parameter directly dependent on the number of OSNs present in the epithelium^45^ (Figure 5C; *Atf5*^+/+^: *N*_mouse_ = 7, *n*_slice_ = 22, 72.4 ± 2.4 μm; *Atf5*^−/−^: *N*_mouse_ = 6, *n*_slice_ = 25, 52.9 ± 2.3 μm; ∗∗∗). Consistent with our observations, and with the previous reports describing a decline in UNO-associated impacts along the nasal cavity,^66^ the *Atf5*^−/−^-associated phenotypes were markedly reduced along the rostral-caudal axis of the MOE (from the dMOE to the dorsal part of the turbinates; Figures 1A and S5A), particularly regarding the proportion of OMP+ neurons and the apoptotic events (Figures S5B–S5D), and they were absent in the GG (Figures 5A and 5B). This strongly suggests that the direct exposure to environmental stimulation is essential for the manifestation of *Atf5* deletion-related defects.

During further investigation of the *Atf5*^−/−^ mice (PM 4–9), we next observed that the basal cell machinery (Figure 2E) was still present to compensate for the massive apoptosis that takes place in the dMOE due the genetic lesion (Figures 5C and 5D). We indeed confirmed the presence of the two basal cell populations involved in neuroepithelium regeneration, the HBCs and the GBCs (Figures S6A and S6B). We used two distinct cellular markers, namely the perinuclear cytokeratin 5 (CK5) targeting HBCs and the nuclear transcription factor sex-determining region Y-box 2 (SOX2) targeting GBCs, which has the peculiarity of persisting in mature supporting cells, the so-called sustentacular cells (Sus; Figures S6A–S6D). Additionally, we observed that the increased apoptotic events occurring in the dMOE of *Atf5*^−/−^ mice had no impact on the sustentacular cells (Figures S6C and S6D) and were restricted to sensory neurons. In parallel, we also observed GAP-43+ immature neurons (Figures 5E and 5F) and Tuj1+ juvenile neurons in both genotypes (Figures S5C and S5D) along the rostral-caudal axis of the MOE (Figure S5A), indicating that *Atf5*^−/−^ OSNs retain the capacity for early differentiation,^22^ independently of environmental stimulation. However, similar to the OMP expression, the signaling elements CNGA2 and ACIII failed to localize to the cilia in *Atf5*^−/−^ OSNs within the dMOE (Figures 5E, 5F, S4C, and S4D), with this defect being dependent on the rostral-caudal axis (Figures S5C and S5D). These findings demonstrate that the loss of ATF5 differentially affects the GG and MOE olfactory subsystems, with the impact being directly dependent on the access to environmental stimulation.

**Figure 6:**
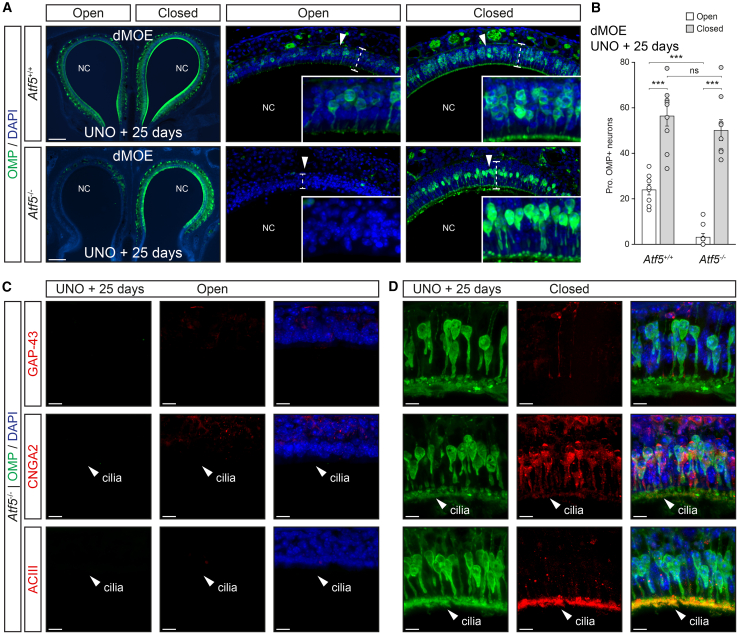
Olfactory neurons with juvenile-like traits are absent in ATF5 knock-out mice (A) Representative immunohistochemistry for the OMP (in green) in the dMOE of *Atf5*^+/+^ (in white) and *Atf5*^−/−^ (in gray) mice after 25 days of UNO. (B) Quantifications of the proportion of OMP+ neurons for the dMOE per genotype and condition (open and closed) are shown. White arrowheads indicate the zoom-in view. White dashed lines designated a representative localization of the epithelium width. (C and D) Double immunohistochemistry for the OMP (in green) and for the GAP-43 or CNGA2 or ACIII (all in red) are illustrated for the open (C) and closed sides (D) from *Atf5*^−/−^ mice. The cilia region (white arrowhead) is indicated to highlight the regular localization of CNGA2/ACIII in (C) and (D). For the morphological aspect, nasal cavities (NC) are indicated in (A). Nuclei are counterstained with DAPI (DAPI, in blue) in (A), (C), and (D) and are used to quantify the proportion (Prop.) of OMP+ neurons according to the number of DAPI+ nuclei in (B). Scale bars represent 200 μm in (A) and 10 μm in (C) and (D). Data are expressed as mean ± SEM with aligned dot plots for ≥5 slices emerging from at least 3 different mice per genotype in (B). For comparisons between conditions and genotypes, two-tailed Student’s t test or Mann-Whitney U test are used, ∗∗∗*p* < 0.001, ns for non-significant in (B).

We then exposed *Atf5*^−/−^ mice (PM 2–6) to increased environmental stimulation (Figures 6A, 6B, S7A, and S7B) and observed an overall and drastic reduction of approximately 85% of OMP+ neurons in the dMOE (Figures 6A, 6B, and 6D; *Atf5*^+/+^: *N*_mouse_ = 3, *n*_slice_ = 8, 24.1 ± 2.4%; *Atf5*^−/−^: *N*_mouse_ = 3, *n*_slice_ = 10, 3.3 ± 1.6%; ∗∗∗), with some tissue slices displaying a complete absence of OMP+ neurons. This was further substantiated by a significant reduction of about 30% in the epithelium width (Figure 6A; open side *Atf5*^+/+^: *N*_mouse_ = 3, *n*_slice_ = 8, 62.7 ± 2.6 μm; open side *Atf5*^−/−^: *N*_mouse_ = 3, *n*_slice_ = 10, 43.7 ± 3.1 μm; ∗∗∗). Interestingly, in the absence of environmental stimulation (closed side), we found similar proportions of OMP+ neurons between genotypes (Figures 6A and 6B; closed side *Atf5*^+/+^: *N*_mouse_ = 3, *n*_slice_ = 9, 56.6 ± 4.6%; closed side *Atf5*^−/−^: *N*_mouse_ = 3, *n*_slice_ = 9, 50.2 ± 4.6%; ns). These OMP+ neurons were predominantly found in the apical region of the epithelium (Figure 6A), suggesting that OMP expression is adapted in OSNs, particularly those potentially exposed to the environment. Moreover, blocking the increased environmental stimulation in *Atf5*^−/−^ mice resulted in a full recovery of the epithelium width (Figure 6A; closed side Atf5^+/+^: *N*_mouse_ = 3, *n*_slice_ = 9, 68.5 ± 3.2 μm; closed side *Atf5*^−/−^: *N*_mouse_ = 3, *n*_slice_ = 9, 66.8 ± 3.1 μm; ns), further supporting the notion of environmental stimulation as a key driver of phenotypic alteration in the dMOE. Interestingly, in heterozygous *Atf5*^+/−^ mice (PM 4), the effects of increased environmental stimulation were intermediate between the ones observed in *Atf5*^+/+^ and *Atf5*^−/−^ mice (Figures S8A and S8B). Specifically, in the rostral part of the dMOE, the stimulated side displayed a partially altered phenotype (Figure S8A) with an approximate 30% reduction in epithelial width between the open and closed sides (Figure S8A; open side *Atf5*^+/−^: *N*_mouse_ = 2, *n*_slice_ = 3, 39.5 ± 5.8 μm; closed side *Atf5*^+/−^: *N*_mouse_ = 2, *n*_slice_ = 3, 61.2 ± 1.7 μm; ∗∗∗). However, this effect gradually diminished along the rostral-caudal axis (Figure S5A), and in the caudo-medial regions, the phenotype became indistinguishable from that of wild-type mice (Figures 6A and 6B), with no reduction in epithelial width between the open and closed sides (Figure 6A; open side *Atf5*^+/−^: *N*_mouse_ = 2, *n*_slice_ = 3, 58.3 ± 2.8 μm; closed side *Atf5*^+/−^: *N*_mouse_ = 2, *n*_slice_ = 3, 60.8 ± 2.4 μm; ns). These findings suggest that heterozygous mice, although showing no apparent defects under standard conditions,^22^ exhibit an increased and regionally constrained sensitivity to heightened environmental stimulation. As expected, such phenomena were not observed in the GG (Figures S7A and S7B). Altogether, these results support the hypothesis that full ATF5 expression is essential for coping with the combined stress of increased sensory input and the requirement for misfolded protein management through the UPR system. They also demonstrate that blocking environmental stimulation is sufficient to rescue the phenotypes associated with *Atf5* genetic deletion observed in the dMOE (Figure 5).

To further investigate the neuronal immaturity induced by environmental stimulations in *Atf5*^−/−^ mice (Figure 6C), we observed that, in the absence of ATF5, environmental stimulation failed to promote the retention of juvenile traits in OSNs. Specifically, there was no increase in GAP-43+ immature neurons in the open side (Figure 6C). Moreover, in line with our previous OMP observations (Figure 6A), blocking the environmental stimulation restored the expression and proper ciliary localization of key signaling elements, such as ACIII, across the sensory epithelium in the closed side (Figures 6D, S4E, and S4F). These results finally demonstrate the causal implication of ATF5 in olfactory neuronal adaptation to environmental stimulation.

## Discussion

Continuous exposure to environmental stimulations is associated with sensory impairment and neurodegenerative diseases that significantly decrease the quality of life.^33^ Olfactory neurons are sentinels of odorant detection and are on the front line of external modifications. Recently, the global pandemic linked to SARS-CoV-2 has highlighted how the loss of this sense (anosmia) impacts our well-being.^33^ However, the intrinsic plasticity of the olfactory system allows it to adapt to the environment.^47^^,^^67^ Neuronal renewal, for example, is a fascinating and exceptional ability that allows the olfactory system to constantly maintain a functional and homeostatic state even in extreme conditions.^68^ However, neuronal renewal has a significant cost in energy and to deal with the constant stimulations linked to the environmental stress, the favored strategy displayed by the developing OSNs is to survive and adapt.

Our research reveals that, in parallel to the well-described sensory desensitization processes occurring at the molecular level in mature OSNs,^25^^,^^49^^,^^69^^,^^70^ immature OSNs may also adapt to increased environmental stimulation by delaying their maturation and stabilizing in a juvenile-like state. This response could represent a protective mechanism that limits potential adverse effects such as neuronal death induced by excessive activity or overstimulation,^71^ which might occur if the neurons reach maturity. It might be particularly relevant in specific situations, such as asymmetric airflow due to nasal obstruction and natural nasal cycles,^15^ or following sudden environmental changes in environmental insults,^19^ where maintaining immature features for a limited time could allow the sensory tissue to temporarily avoid integrating new neurons into a potentially adverse environment. This strategy would help safeguard newly generated neurons and preserve the overall regenerative capacity of the OE, without necessarily impairing global olfactory function. In our study, we chose to extend the observation period to 25 and 50 days in order to improve the visualization of this cumulative cellular phenomenon. These durations allowed for the partial or complete replacement of mature OSNs,^43^ thereby facilitating the detection and analysis of this adaptive process at the tissular level.

From a plasticity perspective, odorant stimulations have been shown to directly modulate OR transcript levels in the MOE, thereby altering the molecular identity of OSNs.^72^^,^^73^^,^^74^ In this context, maintaining OSNs in a juvenile-like state could represent a transient window (Figures S1D–S1F) during which these neurons retain the ability to adapt their expression profile in response to environmental demands. Such plasticity may be particularly relevant for the animal in situations where the odorant landscape undergoes qualitative changes, such as seasonal variations, intense social interactions, or shifts in territory involving novel olfactory cues. In these cases, increased neuroepithelial activity could act as an early signal of impending environmental stress, triggering a delay in terminal maturation that helps preserve the contextual flexibility of OR gene choice. This mechanism, regulated in part by OMP expression,^75^^,^^76^ would thus offer a form of anticipatory flexibility, ensuring that newly maturing OSNs integrate with receptor repertoires better suited to the olfactory environment.

Remarkably, *Atf5*-deficient mice failed to maintain OSNs in a juvenile-like state, suggesting that the loss of ATF5 compromises the ability of newly generated neurons to enter or sustain this protective developmental window. As a result, these neurons may prematurely undergo early differentiation in a context of excessive stimulation, making them more vulnerable to stress-induced apoptosis. Based on our results, beyond its established roles in promoting OSN survival and terminal differentiation,^22^ ATF5 might also serve as a molecular factor, allowing olfactory neurons to delay maturation until environmental conditions become more favorable.

Collectively, our results demonstrate that maintaining juvenile traits in olfactory neurons is an important cellular defense mechanism against unfavorable environments with ATF5 acting as a molecular determinant promoting neuronal adaptation and survival.

### Limitations of the study

Investigating the OSNs juvenile-like phenomenon in the different olfactory subsystems, we observed a direct correlation with the environmental accessibility. The delayed olfactory neuron maturation responses observed in the different olfactory regions (olfactory subsystems and along the rostral-caudal axis of the MOE) were indeed positively correlated with their direct access to the airflow. However, further experiments are still needed to identify the precise environmental stressors triggering this process. Further investigations using, for example, olfactotoxic stimulations,^19^ specific odorant molecules, or modification of physical factors, such as viscosity and temperature,^66^ would allow us to decipher the precise environmental sources involved in this cellular defense that we observed under UNO procedure.

In our experiments, we extended the observation period to 25 and 50 days in order to visualize the global manifestation of the phenomenon at the tissue level. Nevertheless, the emergence of this juvenile-like phenotype at the cellular level is likely to occur rapidly, potentially within a few hours.^15^^,^^16^ However, our experimental design did not allow us to directly investigate such rapid onset. The naris occlusion procedure we used requires anesthesia and post-surgical recovery, preventing the assessment of immediate effects. Alternative approaches, such as reversible plug systems,^77^ may be more appropriate for studying short-term dynamics, and should be considered in future investigations at the cellular level.

In this study, we observed under environmental stimulation (open side) that OSNs in the dMOE no longer express CNGA2 and ACIII in their transduction compartments, suggesting a loss of neuronal function.^78^ Supporting this hypothesis, previous studies using long-lasting UNO paradigms have shown that overexposure to environmental stimuli can reduce olfactory sensitivity in the MOE.^79^ However, we did not perform behavioral or physiological experiments to directly assess whether these molecular changes translate into reduced olfactory sensitivity or hyposmia specifically in the dMOE. To confirm whether the environmental stress-induced shift to a juvenile-like state in OSNs would lead to functional impairment, future studies using *in vivo* or *ex vivo* assays would be valuable.^40^^,^^42^ Such technical approaches could help assess the sensory function of the dMOE and of the GG under environmental stress compared to homeostatic conditions. Moreover, although the classical immaturity markers we used, such as GAP-43 and Tuj1, indicate a general juvenile-like state, they provide only a partial view of the molecular identity of these OSNs. A single-cell transcriptomic approach could enable a more refined characterization of these neurons.^74^ This would help identify the molecular locks induced by environmental stress and determine whether this phenomenon affects specific OSN subpopulations or, as our data suggest, represents a more global effect.

Increased environmental airflow is robust enough to initiate the observed ATF5 expression and related juvenile-like response in OSNs. Nevertheless, we cannot exclude that other triggers may be involved. At birth, for example, we observed that the ATF5 was constitutively expressed in olfactory subsystems where OSNs are naturally challenged by novel external chemical stressors and undergo active neurogenesis, both increasing misfolded protein formation.^60^^,^^61^^,^^62^ In one of the olfactory subsystems, the GG, these cumulative effects, restricted to the perinatal stage, are also associated with the transient expression of ORs^80^ that could thus also contribute to the time-restricted ATF5 nuclear expression.

In our study, we used constitutive *Atf5* knockout mice, which do not allow for the precise dissection of ATF5 specific roles at particular developmental stages or in distinct cell types within the OE. Although our findings support the idea that ATF5 contributes to maintain newly generated OSNs in an immature state in response to increased stimulation, we cannot exclude the possibility that some of the observed effects result from broader developmental disruptions caused by the constitutive loss of *Atf5*. A conditional deletion approach targeting *Atf5* specifically in mature OSNs, for instance using an OMP-Cre line,^81^ would help detail the ATF5 role in this regulation. Such an approach could determine whether ATF5 expression in immature neurons is sufficient to preserve their juvenile characteristics under a UNO paradigm, while its absence in mature neurons could lead to their selective loss. While these targeted strategies lie beyond the scope of the present study, they would represent an important step toward a better characterization of ATF5 functions according to cellular context and developmental stage in the olfactory system.

Furthermore, it would be interesting to study the conservation of a juvenile-like state linked to activity beyond the olfactory system. Indeed, similar phenomena have been observed in other sensory epithelia,^10^ where the level of stimulation modulates neuronal maturation. Comparing the responses of the olfactory system to those of other sensory systems could help identify common mechanisms of plasticity and control of neuronal development in the context of overstimulation and environmental stress.

## Resource availability

### Lead contact

Requests for further information and resources should be directed to and will be fulfilled by the lead contact, Marie-Christine Broillet (marie-christine.broillet@unil.ch).

### Materials availability

The constructs for *Atf5* null mice generated in this study are available from the lead contact upon request.

### Data and code availability

• Microscopy data reported in this paper will be shared by the lead contact upon request.
• This study does not report original code.
• Any additional information required to reanalyze the data reported in this paper is available from the lead contact upon request.

## Acknowledgments

The authors thanks M. Auberson and C. Verdumo for their valuable advices and assistance during RT-PCR and RT-qPCR experiments; I. Rodriguez for the OMP-GFP mice and G. Cretenet and C. Bolvin who initiated the *Atf5* mouse model and the Institut Clinique de la Souris from the Institute of Genetics and Molecular and Cellular Biology of Strasbourg for the generation of ATF5 KO mice; A. Scapuso and B. Pilet for their technical support during the histological investigations and the mice genotyping as well as the animal facility of the department for the animal care; I. Rodriguez and A. Ocampo for fruitful discussions on the experimental results; and the Cellular Imaging Facility of the University of Lausanne and his coordinator J.-Y. Chatton. We acknowledge BioRender.com for providing tools to create schematic illustrations. This work was supported by the Réseau National des Génopoles: IR1314/K379 (to F.G.), the Department of Biomedical Sciences of the University of Lausanne (to M.-C.B.), and by the 10.13039/501100001711Swiss National Science Foundation Grant 310030_185161 (to M.-C.B.).

## Author contributions

J.B., F.G., and M.-C.B. designed the project. F.G. provided the *Atf5* mouse line. Data collection and investigations were performed by J.B., A.C.L., D.W., E.S., M.S., N.G., and M.N.T. J.B. and A.C.L. analyzed the data. J.B. and M.-C.B. supervised the project and wrote the original draft of the manuscript.

## Declaration of interests

The authors declare no competing interests.

## STAR★Methods

### Key resources table

**Table:** 

REAGENT or RESOURCE	SOURCE	IDENTIFIER
**Antibodies**		

Rabbit polyclonal anti-GAP-43	Novus	Cat# NB300-143; RRID:AB_10001196
Mouse monoclonal anti-beta III Tubulin (Tuj1)	Abcam	Cat# ab78078 [2G10]; RRID:AB_2256751
Rabbit polyclonal anti-Adenylate Cyclase 3	Novus	Cat# NBP1-92683; RRID:AB_11031204
Rabbit polyclonal anti-CNGA2	Alomone Labs	Cat# APC-045; RRID:AB_2039820
Rabbit polyclonal anti-ATF-5 [H-83]	Santa Cruz Biotechnology	Cat# sc-99205; RRID:AB_2058755
Rabbit polyclonal anti-ATF5	Abcam	Cat# ab60126; RRID:AB_940375
Rabbit polyclonal anti-pGCG	Fabgennix	Cat# PGCG-701AP; RRID:AB_2315124
Goat polyclonal anti-OMP	FUJIFILM Wako Pure Chemical Corporation	Cat# 544-10001-WAKO; RRID:AB_664696
Rabbit polyclonal anti-PDE2A	Fabgennix	Cat# PD2A-101AP; RRID:AB_2315082
Rabbit polyclonal anti-Cleaved Caspase-3 [Asp175]	Cell Signaling Technology	Cat# 9661; RRID:AB_2341188
Rabbit polyclonal anti-SOX2	Thermo Fisher Scientific	Cat# PA1-094; RRID:AB_2539862
Rabbit monoclonal anti-CK5	Abcam	Cat# ab52635; RRID:AB_869890
Cy3-conjugated, goat polyclonal anti-Rabbit	Jackson ImmunoResearch Labs	Cat# 111-165-144; RRID:AB_2338006
Cy3-conjugated, donkey polyclonal anti-Rabbit	Jackson ImmunoResearch Labs	Cat# 711-165-152; RRID:AB_2307443
Alexa Fluor Plus 647-conjugated, goat polyclonal anti-Mouse	Thermo Fisher Scientific	Cat# A32728; RRID:AB_2633277
Alexa Fluor 568-conjugated, donkey polyclonal anti-Mouse	Thermo Fisher Scientific	Cat# A10037, RRID:AB_11180865
FITC-conjugated, rabbit polyclonal anti-Goat	Jackson ImmunoResearch Labs	Cat# 305-095-003; RRID:AB_2339439
FITC-conjugated, donkey polyclonal anti-Goat	Jackson ImmunoResearch Labs	Cat# 705-095-147; RRID:AB_2340401

**Biological samples**		

Mouse olfactory tissues (GG; dMOE; MOE; VNO; SO)	This study	N/A
Mouse brain tissue	This study	N/A
Mouse heads	This study	N/A

**Chemicals, peptides, and recombinant proteins**		

Cyanoacrylate glue	Carl Roth	Cat# CA221
Paraformaldehyde	Sigma-Aldrich	Cat# 158127
Ethylenediaminetetraacetic acid disodium salt dihydrate (EDTA)	Sigma-Aldrich	Cat# 03685
Agar (low-melting)	Sigma-Aldrich	Cat# A7002
Normal goat serum	Jackson ImmunoResearch	Cat# 005-000-121
Normal rabbit serum	Jackson ImmunoResearch	Cat# 011-000-120
Normal donkey serum	Jackson ImmunoResearch	Cat# 017-000-121
Triton™ X-100	Sigma-Aldrich	Cat# 93420
Chromium(III) potassium sulfate dodecahydrate	Sigma-Aldrich	Cat# 60151
Gelatin from porcine skin	Millipore	Cat# 48722

**Critical commercial assays**		

VECTASHIELD® Antifade Mounting Medium with DAPI	Vector Laboratories	Cat# H-1200
5-Bromo-2′-deoxy-uridine Labeling and Detection Kit I	Roche	Cat# 11296736001
RNeasy Plus Mini Kit	Qiagen	Cat# 74134
GoTaq® DNA Polymerase	Promega	Cat# M3001
Fast SYBR Green Master Mix	Thermo Fisher Scientific	Cat# 4385617
PrimeScript™ 1st strand cDNA Synthesis Kit	Takara	Cat# 6110A

**Deposited data**		

Original data for immunohistochemistry and confocal acquisitions	This study	N/A
Original data for statistical analysis	This study	N/A

**Experimental models: Organisms/strains**		

Mouse: B6;129P2-Omp^tm3Mom^/MomJ	The Jackson Laboratory	RRID:IMSR_JAX:006667; MGI:J:73384
Mouse: *Atf5* null mice (*Atf5*^-/-^)	This study	N/A

**Oligonucleotides**		

Primers for PCR, see Table S1	This study	N/A
Primers for RT-PCR, see Table S1	This study	N/A
Primers for RT-qPCR, see Table S1	This study	N/A

**Software and algorithms**		

Imaris v7.1.1	Bitplane	RRID:SCR_007370; https://imaris.oxinst.com/packages
ImageJ v1.53a	National Institute of Health	RRID:SCR_003070; https://imagej.net
GraphPad Prism v9.1.1	GraphPad Software Inc.	RRID:SCR_002798; https://www.graphpad.com

**Other**		

Dual Temperature Cautery Kit (Gemini Cautery Kit)	Stoelting	Cat# 59017
Fluorescent Stereo Microscope	Leica Microsystems	Cat# M165 FC
LED illuminator for microscopes pE-300^white^	CoolLED	Cat# pE-300-W-D-SB

### Experimental model and study participant details

Male and female mice were equally used for all experiments, with no observed influence of sex on the results. Pups and adult mice of corresponding ages were used for comparisons between genotypes and conditions, and are indicated as follows: postnatal day (P) for pups, and postnatal month (PM) for adults. Heterozygous mice from OMP-GFP (B6;129P2-Omp^tm3Mom^/MomJ) gene-targeted mouse strain^33^^,^^40^ were used. In this mouse line, the GFP is fused with the TAU protein, expressed under the control of the OMP promoter^51^^,^^52^ and is used as a histological reporter of mature olfactory sensory neurons.^34^ The observed GFP-related signal is mentioned as *tau*GFP. For the generation of *Atf5* null mice, the Cre-LoxP strategy was used to delete the exon 3 containing the bZip domain. For the construction of the *Atf5* targeting vector, a 4.3 kb fragment containing exons 1 and 2 served as the 5’ arm, and a 3.2 kb fragment containing genomic sequences after exon 3 served as the 3' arm. These two fragments were cloned upstream and downstream, respectively, of a modifying allele containing exon 3 flanked by 2 LoxP sites and a PGK-Neo cassette containing a LoxP site and flanked by 2 FRT sites. The resulting targeting vector was electroporated into BD10 [MCI-C57BL/6N] ES cells. One selected positive clone was microinjected into blastocysts, and one chimera strain with a transmissible allele was selected. The PGK-Neo cassette was removed by crossing these mice with ATCB-Flp mice^82^ to obtain the conditional knockout allele. This mouse strain was finally crossed with CMV-Cre mice^83^ to obtain the KO allele and *Atf5* null mice and backcrossed to C57BL/6J (B6). Heterozygous *Atf5* mice (*Atf5*^+/-^) were used for breeding to generate littermates of different genotypes (*Atf5*^+/+^, *Atf5*^+/-^ and *Atf5*^-/-^). Mice were housed in the animal facility under 12:12 light-dark cycle. Sacrifices were performed under CO_2_ or cervical dislocation. The experimental procedures were in accordance with the Swiss legislation and approved by the EXPANIM committee of the Lemanique Animal Facility Network and the veterinary authority of the Canton de Vaud (SCAV; VD2496.2, VD2496.3, VD2496.x3).

### Method details

#### Genotyping

Alkaline extraction of mouse DNA followed by polymerase chain reaction (PCR) procedures were used to amplified specific alleles.^84^ PCRs were conducted in a thermocycler (Veriti™; Applied Biosystems) with the primers listed in the Table S1. The 100 bp-DNA ladder (Bench Top G829B; PROMEGA) and the PCR products were then loaded on an 3% agarose gel supplemented with ethidium bromide under 80 V-electrophoresis. DNA amplicon sizes were revealed under UV light.

#### Unilateral naris occlusion (UNO)

To investigate how environmental stimulation impacted the olfactory system, we performed UNO, a well-established method that induced sensory deprivation on the “Closed” side and a sustained stimulation on the contralateral “Open” side, thereby creating a controlled asymmetry of olfactory input.^69^^,^^85^ The procedure was adapted from previously published protocols.^19^^,^^45^^,^^86^ Briefly, under a dissecting stereomicroscope (M80, Leica), the procedure was carried out on deeply anesthetized mice^87^ at the indicated ages. A low-temperature cauterization device (Gemini Cautery Kit, Stoelting) was applied to seal one naris (left or right naris equally performed). To ensure complete closure and prevent reopening, the external skin was sealed using cyanoacrylate glue (CA22; Carl Roth). Animals were kept on a heating pad (37°C) during recovery, then returned to their home cage. To validate successful occlusion, we inspected the absence of air bubbles from the occluded naris when a drop of phosphate-buffered saline (PBS; 138 mM NaCl, 2.7 mM KCl, 1.76 mM KH_2_PO_4_, and 10 mM Na_2_HPO_4_, pH 7.4) was applied, indicating lack of airflow. Mice were monitored daily to assess overall development and to detect any signs of spontaneous reopening of the occluded naris. In cases where the closure was maintained for at least 25 days, we observed that reopening of the naris led to a recovery of *tau*GFP signals to baseline levels within approximately five days, supporting the reversibility of the procedure and confirming the high degree of OSN plasticity.^49^ Otherwise mentioned, animals in which occlusion was incomplete or reversed were excluded from analysis.

#### Neuronal maturity level inspection by *tau*GFP expression profile

Dissections of the mouse heads were performed under ice-cold sterile PBS conditions.^33^ To allow the epi-visualization of the olfactory systems (GG, dMOE and OB), the skin was delicately removed and mouse heads were individually placed in 4% paraformaldehyde fixative solution (PAF 4%, pH 7.4) for 24 hours at 4°C. After washing steps, whole-mount views of mouse heads were then placed under a fluorescent stereomicroscope (M165 FC; Leica), their observations were realized under specific GFP illumination (pE-300white Series; CoolLED) and their acquisitions were performed with a sensitive camera (MC170 HD; Leica). For slice view observations, individual whole-mount preparations were then transferred for 24 hours in a 0.5 M EDTA (Ethylenediaminetetraacetic acid, pH 8.0) decalcifying solution. GG and the dMOE were then collected and placed in a 4% low-melting agar block (A7002; Sigma-Aldrich). Serial coronal tissue slices of respectively 80 μm and 120 μm were then generated with a vibroslicer (VT1200S; Leica) and collected in ice-cold PBS. After background subtraction, the *tau*GFP intensities (GFP-related signals) were then quantified with ImageJ software (v1.53a; National Institute of Health). An average of a minimum of three slices was used for the establishment of the global *tau*GFP intensity values per olfactory subsystem/condition and a minimum of 5 animals were used per subsystem/condition.

#### Quantification of the cellular proliferation

The cellular proliferation level was assessed through 5-Bromo-2’-deoxy-uridine (BrdU) DNA labeling, following the manufacturer's instructions (5-Bromo-2’-deoxy-uridine Labeling and Detection Kit 1; Roche). To avoid cellular compensations limited to the surgical procedure recovery (few days) and/or to subtype-specific OSNs,^5^^,^^47^ mice were treated with BrdU injections 15 days post-naris occlusion procedure. Injections were done four times daily with 0.5 ml / 100 g body weight of sterile BrdU [10 mM] labeling reagent. Subsequently, mice were euthanized by cervical dislocation, and the dissected dMOE was promptly frozen using liquid propane. Cryosections of 20 μm thickness were then generated using a cryostat (HM525 NX; Thermo Fischer Scientific) on chromium-gelatin-coated microscope slides (SuperFrozt; Menzel-Gläser). Cryosections were then post-fixed for 20 minutes at -20°C using a 50 mM EtOH-glycine solution at pH 2.0. Detection was performed using a nuclease-implemented mouse anti-BrdU reagent and a sheep anti-mouse Fluorescein solution. Nuclei were counterstained with DAPI using antifade mounting medium (Vectashield®; H-1200; Vector Labs). A minimum of two cryosections per animal were acquired by confocal microscopy (SP5; Leica). Maximum projections, reconstructions, and analyses were performed using computer assistance (v7.1.1, Imaris; Bitplane). The density of proliferating cells was then quantified with ImageJ software (v1.53a; National Institute of Health) and expressed as the number of BrdU+ nuclei per μm^2^.

#### Protein expression profile

The expression and the precise localization of proteins were investigated by immunostainings.^33^^,^^88^ Briefly, after an initial 24 hours of PAF 4% fixation, mouse heads were individually transferred in a 0.5 M EDTA decalcifying solution for 24 hours. Precise dissections of the GG, dMOE and OB were then performed in ice-cold PBS and the obtained tissues were included in a 4% low-melting agar block (A7002; Sigma). For floating sections, serial coronal slices of respectively 80 μm, 120 μm and 100 μm were then generated with a vibroslicer (VT1200S; Leica) and collected in ice-cold PBS. Specific investigations along the rostral-caudal axis of the MOE were performed on 50 μm cryosections using a cryostat (HM 525NX; Thermo Fisher Scientific). Selected slices were then blocked and permeabilized in a PBS solution containing 10% of normal goat serum (NGS; 005-000-121; Jackson ImmunoResearch), 5% of normal rabbit serum (NRS; 011-000-120; Jackson ImmunoResearch) or 5% of normal donkey serum (NDS; 017-000-121; Jackson ImmunoResearch) depending on the conditions and all supplemented with 1% Triton X-100 (TX100; Fluka analytical) for at least 24 hours at 4°C. Then, an indirect immunostaining approach was used with the primary antibodies listed in the key resources table: GAP-43 (Rabbit polyclonal anti-GAP-43; NB300-143; Novus; 1:1000); Tuj1 (Mouse monoclonal anti-beta III Tubulin; ab78078 [2G10]; Abcam; 1:650); ACIII (Rabbit polyclonal anti-Adenylate Cyclase 3; NBP1-92683; Novus; 1:2000); CNGA2 (Rabbit polyclonal anti-CNGA2; APC-045; Alomone Laboratories; 1:200); ATF5 (Rabbit polyclonal anti-ATF-5 [H-83]; sc-99205; Santa Cruz; 1:100; if not mentioned | Rabbit polyclonal anti-ATF5; ab60126; abcam; 1:150; mentioned); GC-G (Rabbit polyclonal anti-pGCG; PGCG-701AP; FabGennix; 1:250); OMP (Goat polyclonal anti-OMP; 544-10001-WAKO; FUJIFILM Wako Pure Chemical Corporation; 1:1000); PDE2A (Rabbit polyclonal anti-PDE2A; PD2A-101AP; FabGennix; 1:400); CASP3 (Rabbit polyclonal anti-Cleaved Caspase-3 [Asp175]; 9661; Cell signaling; 1:2000); SOX2 (Rabbit polyclonal anti-SOX2; PA1-094; Thermo Fisher Scientific; 1:400) and CK5 (Rabbit monoclonal anti-CK5; ab52635; Abcam; 1:100). Detection of primary antibodies was performed using fluorochrome-conjugated secondary antibodies listed in the key resources table against rabbit (Cy3-conjugated, goat polyclonal anti-Rabbit; 111-165-144; Jackson ImmunoResearch; 1:200), or (Cy3-conjugated, donkey polyclonal anti-Rabbit; 711-165-152; Jackson ImmunoResearch; 1:200), against mouse (Alexa Fluor Plus 647-conjugated, goat polyclonal anti-Mouse; A32728; Thermo Fisher Scientific; 1:200), or (Alexa Fluor 568-conjugated, donkey polyclonal anti-Mouse; A10037; Thermo Fisher Scientific; 1:200), as well as against goat (FITC-conjugated, rabbit polyclonal anti-Goat; 305-095-003; Jackson ImmunoResearch; 1:200), or (FITC-conjugated, donkey polyclonal anti-Goat; 705-095-147; Jackson ImmunoResearch; 1:200). Slices were then rinsed in 2% of respective serum solution and nuclei were counterstained with DAPI thanks to the antifade mounting medium (Vectashield®; H-1200; Vector Labs). Negative controls were systematically performed by omitting primary antibodies. Initial observations were made by LED-fluorescence microscopy (EVOS M5000; Invitrogen) and final acquisitions were performed with confocal microscopy (SP5 without mention or Stellaris 8; Leica). Maximum projections, reconstructions and analysis were made with computer assistance (v7.1.1, Imaris; Bitplane). When required, the cellular density, the cellular proportion and the protein expression profile were quantified with ImageJ software (v1.53a; National Institute of Health). The cellular density was obtained by the number of DAPI+ nuclei / μm^2^ and the cellular proportion was obtained by the number of specific marker+ cells / DAPI+ nuclei counted in the same surface (for DAPI+ cells, no distinction was made between neuron, glial or other cellular types). A minimum of two slices per olfactory subsystem were used per condition and animal. The protein expression profile across the olfactory epithelium was obtained by cumulating measurements from a minimum of 5 different sections per condition.

#### Gene expression profile

RNA purification procedure^33^ was achieved according to the manufacturer instructions (RNeasy® Plus Mini kit; Qiagen). GG, OE (mix of MOE, dMOE and SO epithelia) or dMOE samples were obtained from 2-4 mice at the indicated age and pooled in buffer RLT Plus supplemented with β-mercaptoethanol. High-speed tissue disruption (TissueLyser II; Qiagen) was performed for sample homogenization. The genomic DNA of the lysate was removed by using the gDNA Eliminator spin column. RNeasy spin columns were then used for centrifugation processes and the final total RNA eluates were collected in 30 μl of RNase-free water. The random hexamers option of the cDNA synthesis kit (PrimeScript™ 1st strand cDNA Synthesis Kit; Takara) was then used to initiate the reverse transcription (RT) procedure using 140 ng of RNA to finally obtain 20 μl of cDNA.

For real-time polymerase chain reaction^33^ (RT-PCR), PCR experiments were performed using 3 μl of cDNA as a template. The specific primers, built-in exon-exon boundaries design using the Primer3plus resource, at a concentration of 800 nM were used and are listed in the Table S1: *Atf5* (product size of 175 bp) and *Gapdh* (product size of 223 bp). Amplifications were done using 1.0 unit of DNA polymerase (GoTaq® DNA Polymera; Promega) for 30 cycles in a thermocycler (Veriti™; Applied Biosystems) programmed at 95°C 30 sec, 55°C 30 sec and 68°C 45 sec. After 80V-electrophoresis on a 3% agarose gel supplemented with ethidium bromide, amplica were then visualized under UV light and their sizes were assessed (Bench Top 100bp DNA Ladder; Promega). Negative controls were performed by omitting the reverse transcription phase (-RT) or with H_2_O. The RT-PCR semi-quantitative analyses^33^ were calculated with ImageJ software (v1.53a; National Institute of Health) according to the intensities detected under the curves relatively expressed to *Gapdh*.

For quantitative RT-PCR (RT-qPCR), Real-Time PCR system detector (7500 Fast Real-Time PCR System; Applied Biosystems) was used for the amplification of the *Atf5* transcript (product size of 175 bp), the housekeeping gene *Gapdh* (product size of 223 bp) according to specifics primers listed in the Table S1 as well as for the internal control H_2_O. Duplicates were performed with a final volume of 20 μl. All reactions were generated with 1x SYBR® Green enzyme (Fast SYBR Green Master Mix; Thermo Fisher Scientific), 800 nM of primers, 3 μl of cDNA and by using universal and fast PCR cycling conditions. A limit threshold of 0.15 was applied to compare the different C_T_ for further analysis. The RNA fold-change normalized to *Gapdh* was calculated according to the comparative 2^(-ΔΔCT)^ method and melting curve analysis was used to confirm specificity of amplification.^33^^,^^89^

### Quantification and statistical analysis

GraphPad Prism v9.1.1 was used for statistical analysis and the generation of aligned dot-plot graphics. Values are expressed as mean ± standard error of the mean (SEM). Sample size was determined according to previous publications and based on pilot experiments. Shapiro and Fisher *F*-tests were used for the evaluation of normality and homoscedasticity. Comparisons were then performed with two-tailed unpaired Student’s *t*-test (with Welch’s correction in absence of homoscedasticity) or Mann-Whitney *U*-tests accordingly. Significance levels are indicated as follows: ∗ p<0.05, ∗∗ p<0.01, ∗∗∗ p<0.001 and ns for non-significant.
