# Supplementary material for: Environmental stress promotes the persistence of juvenile traits in olfactory neurons as a protective mechanism
Source: iScience. 2025 Jul 24;28(8):113078. doi: 10.1016/j.isci.2025.113078 (PMC12432459; doi:10.1016/j.isci.2025.113078)
Supplement: Document S1. Figures S1–S8 and Table S1 [file mmc1.pdf]

iScience, Volume *28*

Supplemental information

Environmental stress promotes the persistence of juvenile traits in olfactory neurons as a protective mechanism

Julien Brechbühl, Ana Catarina Lopes, Dean Wood, Elodie Sauge, Marianne Sidhom, Noah Gilliand, Monique Nenniger Tosato, Frédéric Gachon, and Marie-Christine Broillet

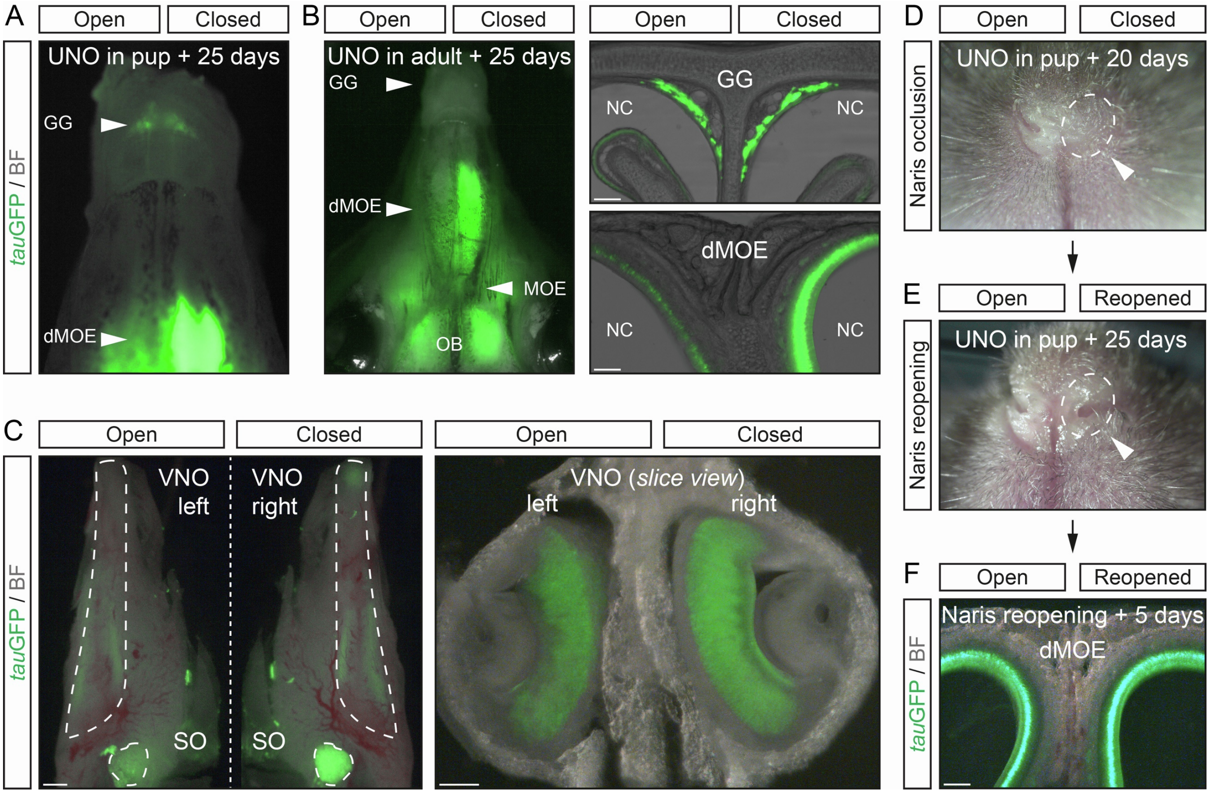

Figure S1. Increased environmental stimulations differentially impact *tau*GFP signal across olfactory subsystems. Related to Figure 1.

(A-C) Visualization of the *tau*GFP signal (in green) 25 days post-UNO in the different olfactory subsystems, the Grueneberg ganglion (GG), the main olfactory epithelium (MOE) and its dorsal part (dMOE), the vomeronasal organ (VNO) and the septal organ of Masera (SO). High power view of the dashed white rectangle from the Figure 1B in (A). 25 days post-UNO performed on an adult heterozygote OMP-GFP mouse observed in Whole-mount (left panel of (B)) and coronal Slice view (right panels of (B)). Whole-mount view of VNO and SO (left panel of (C)). Coronal Slice view of VNO (right panels of (C)).

(D-F) The naris reopening procedure. Inspection of the UNO efficiency in *en face* views after 20 (D), and 25 (E) days on a heterozygous OMP-GFP mouse. After 25 days the mouse intervention reopened the previously Closed naris (E). The naris reopening (Reopened) is highlighted with a dashed line and white arrowhead (UNO original localization). 5 days after the naris reopening (F), the *tau*GFP signal in the dMOE is restored. Bright-field illumination (BF) in (A), (B) and (C). For the morphological aspect, nasal cavities (NC) and olfactory bulb (OB) are indicated in (A), (B) and (C). Scale bars are 100 μm in (B) and (F), 500 μm in the Whole-mount view of (C), 200 μm in the Slice view of (C).

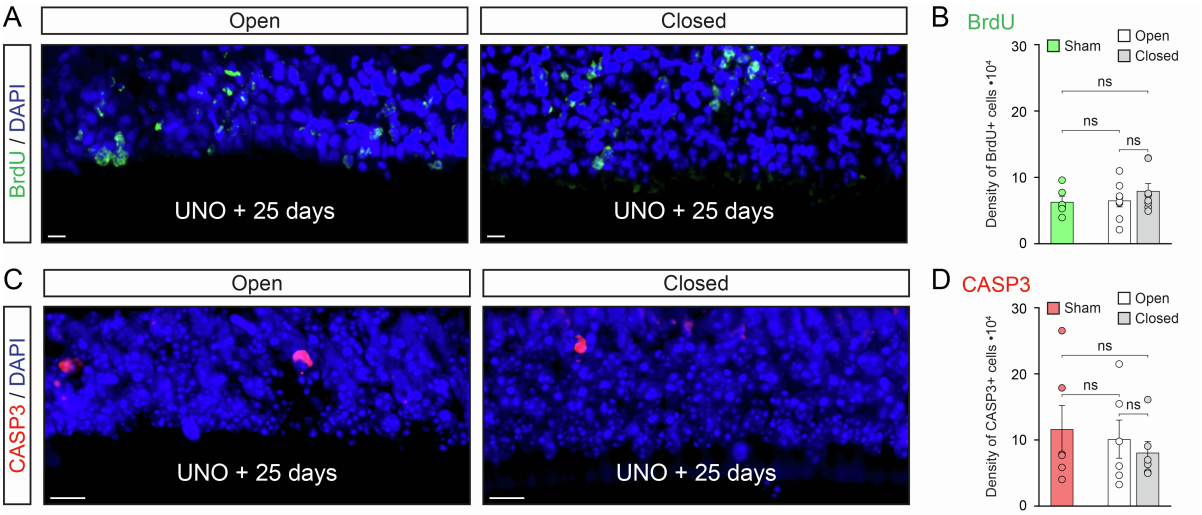

Figure S2. Cellular proliferation and apoptosis are not affected by increased environmental stimulations. Related to Figures 1 and 2.

(A) At 25 days post-UNO, neosynthetized cells are estimated thanks to the BrdU proliferation assay (in green).

(B) The quantification of BrdU+ cells reveals the absence of significant difference between Open (in white) and Closed (in gray) sides as well as to Sham-operated mice (in green).

(C) Apoptosis is estimated thanks to the CASP3 staining (in red).

(D) The quantification of CASP3+ cells reveals the absence of a significant difference between the Open (in white) and the Closed (in gray) sides as well as with the Sham-operated mice (in red). Scale bars are 10 μm in (A) and (C). Data are expressed as mean ± SEM with aligned dot plots for ≥ 6 mice in (B) and (D). For comparisons between conditions, two-tailed Student's t-test or Mann-Whitney U-test are used, ns for non-significant in (B) and (D).

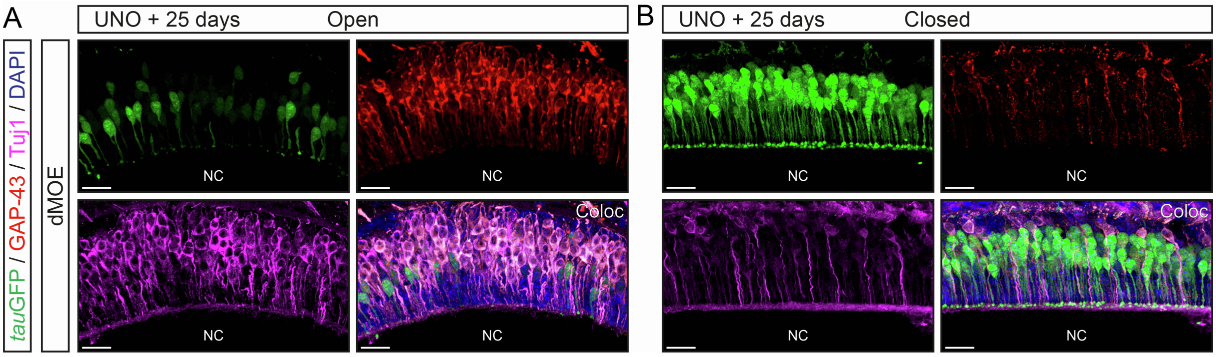

Figure S3. Increased environmental stimulations promote the presence of GAP-43/Tuj1+ OSNs in the dMOE. Related to Figure 2.

(A and B) Double immunohistochemistry for the markers of immature/juvenile neurons, respectively the growth-associated protein 43 (GAP-43, in red), and the neuron-specific class III β-tubulin (Tuj1, in purple) on heterozygous OMP-GFP mice, after 25 days of UNO. Representative immunostainings from the dMOE are shown for the Open (A) and the Closed (B) side. The *tau*GFP signal (in green), and the GAP-43/Tuj1 colocalization signal (Coloc, in white) are shown. For the morphological aspect, nasal cavities (NC) are indicated in (A) and (B). Nuclei are counterstained with dapi (DAPI, in blue) in (A), and (B). Scale bars are 20 μm in (A), and (B). Confocal acquisitions were made with Leica Stellaris 8 in (A), and (B).

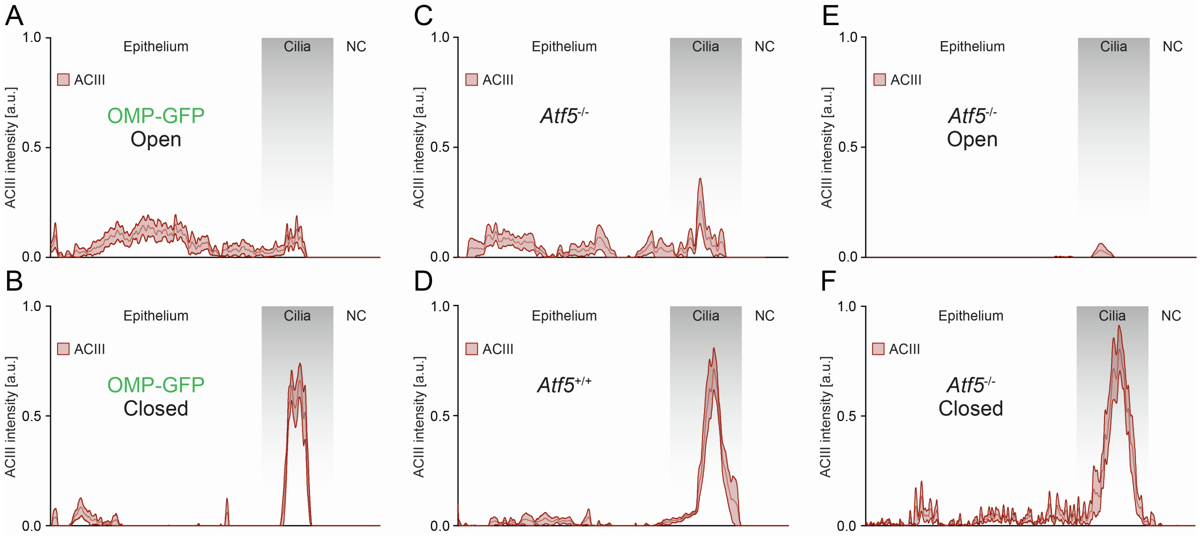

Figure S4. ACIII ciliary targeting is influenced by increased environmental stimulations and by ATF5. Related to Figures 2, 5 and 6.

(A-F) Expression profile of the ACIII (in red lines) across the sensory epithelium of the dMOE for the following genotypes, OMP-GFP in (A) and (B), *Atf5*$^{+/+}$ in (D) and *Atf5*$^{-/-}$ in (C), (E) and (F) under the indicated conditions: Closed in (B) and (F), Open in (A) and (E) or with no naris occlusion procedure in (C) and (D). The cilia region is highlighted in the gray rectangle in between the epithelium and the nasal cavity (NC). Curves are obtained from the average of 10 independent intensity measurements ± SEM in (A-F).

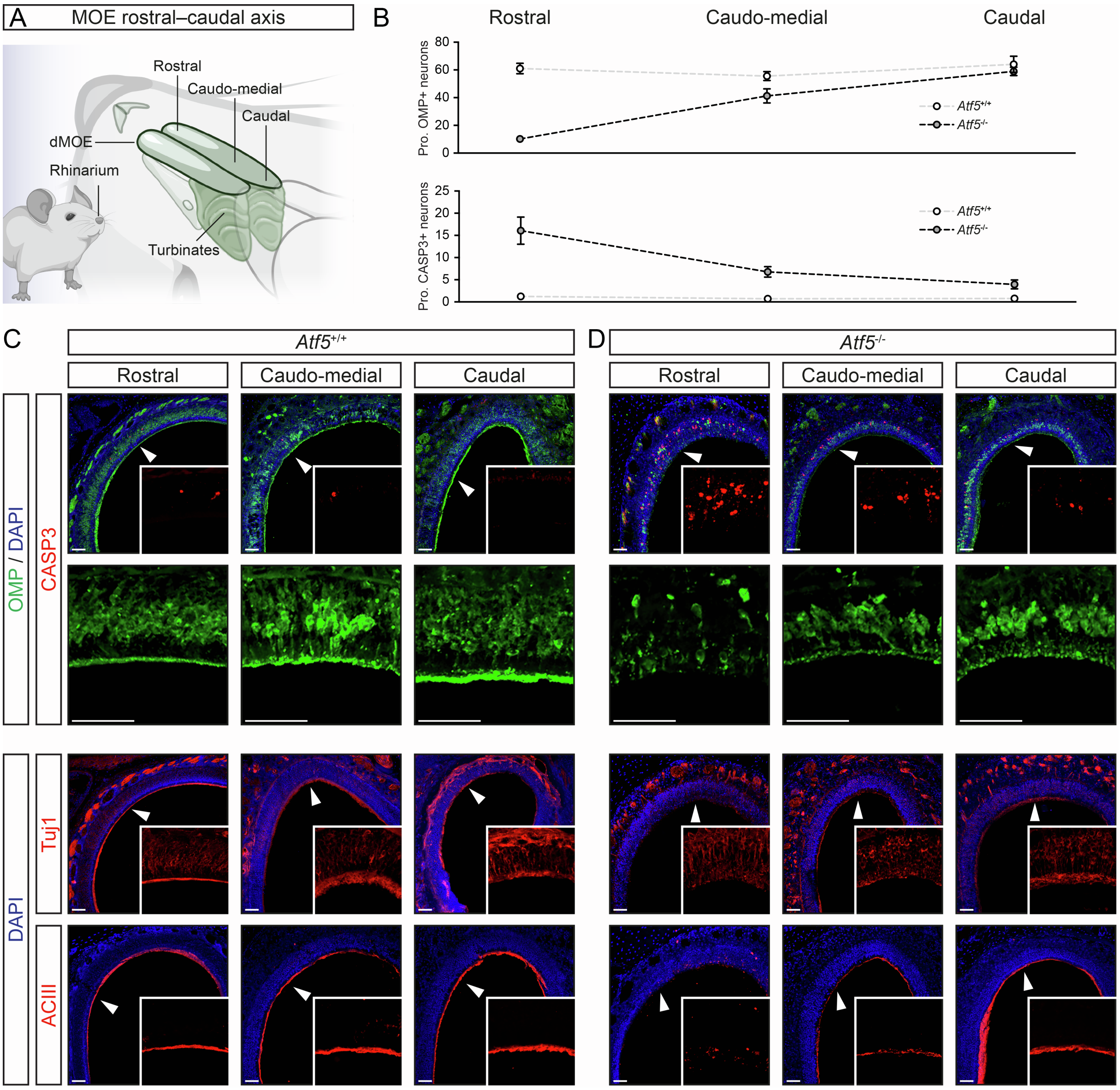

Figure S5. *Atf5*$^{-/-}$-associated phenotypes depend of the rostral-caudal axis of the MOE. Related to Figure 4.

(A) Schematic representation of a mouse head illustrating the rostral, caudo-medial, and caudal regions along the rostral-caudal axis of the MOE. The dMOE, turbinates, and the location of the rhinarium are also indicated.

(B) Quantifications of the proportion (Pro.) of OMP+ neurons (upper panel) and CASP3+ neurons (lower panel) along the rostral-caudal axis in *Atf5*$^{+/+}$ (white) and *Atf5*$^{-/-}$ (gray) mice.

(C-D) Representative images along the rostral-caudal axis for *Atf5*$^{+/+}$ (C) and *Atf5*$^{-/-}$ (D) mice showing OMP (green), CASP3, Tuj1, and ACIII (all in red). Nuclei are counterstained with DAPI (blue) in both (C) and (D), and used to calculate the proportion of OMP+ and CASP3+ neurons relative to the total number of DAPI+ nuclei as shown in (B). Scale bars: 50 μm in (C) and (D). Data in (B) are presented as mean ± SEM for ≥ 5 sections from 2 different mice per genotype. Confocal acquisitions of the cryosections were made with Leica Stellaris 8 in (C) and (D).

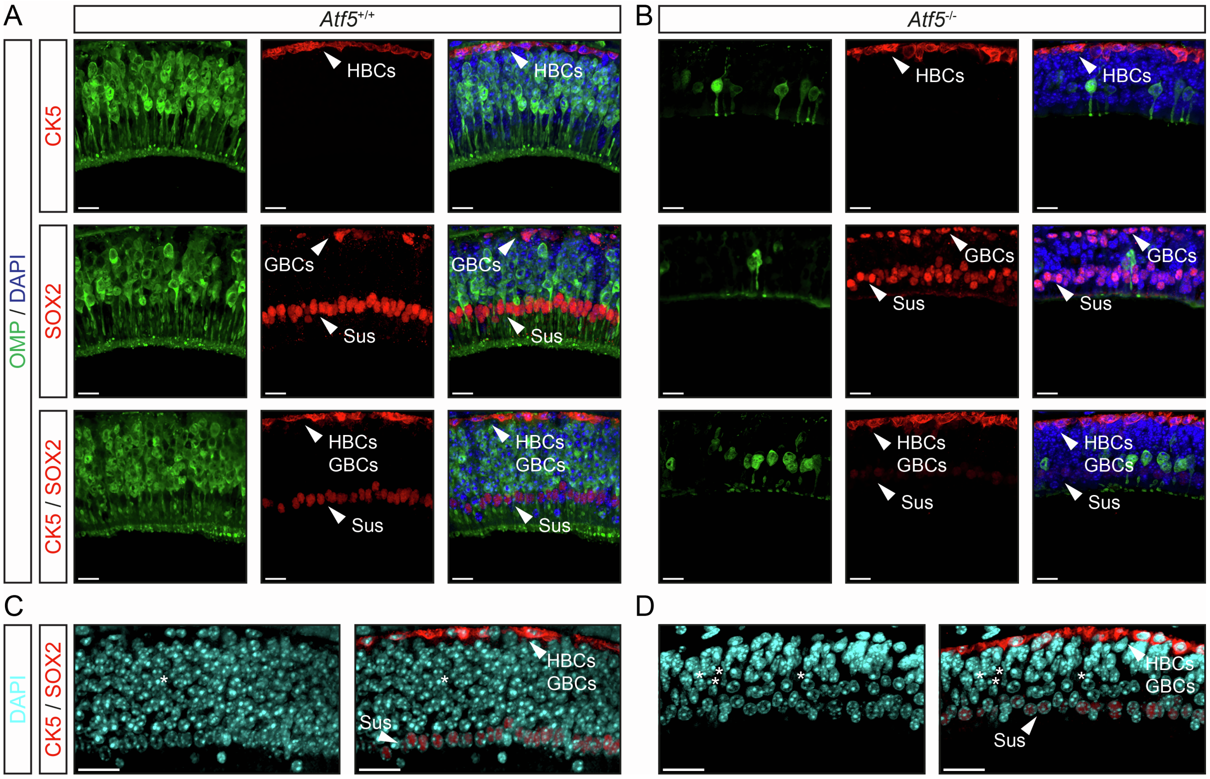

Figure S6. Basal cell machinery and sustentacular cells are present in the dMOE of *Atf5*$^{-/-}$ mice. Related to Figure 5.

(A and B) Double immunohistochemistry for the OMP (in green) and for the CK5, the SOX2 or both CK5 / SOX2 (all in red) are illustrated for *Atf5*$^{+/+}$ in (A) and *Atf5*$^{-/-}$ (B) mice. CK5+ horizontal basal cells (HBCs), SOX2+ globose basal cells (GBCs), and SOX2+ mature sustentacular cells (Sus) are indicated (white arrowheads).

(C and D) Detailed of CK5 / SOX2 staining from (A) and (B) here used to observe the apoptotic events according to pyknotic nuclei (indicated by white asteria). CK5 / SOX2 labeling (in red) is used to differentiate sustentacular cells and basal cells in the epithelium of *Atf5*$^{+/+}$ in (C) and *Atf5*$^{-/-}$ in (D) mice. Nuclei are counterstained with dapi (DAPI, in blue) in (A) and (B) and cyan in (C) and (D). Scale bars are 15 μm in (A) and (B) and 10 μm in (C) and (D). Confocal acquisitions were made with Leica Stellaris 8 in (A-D).

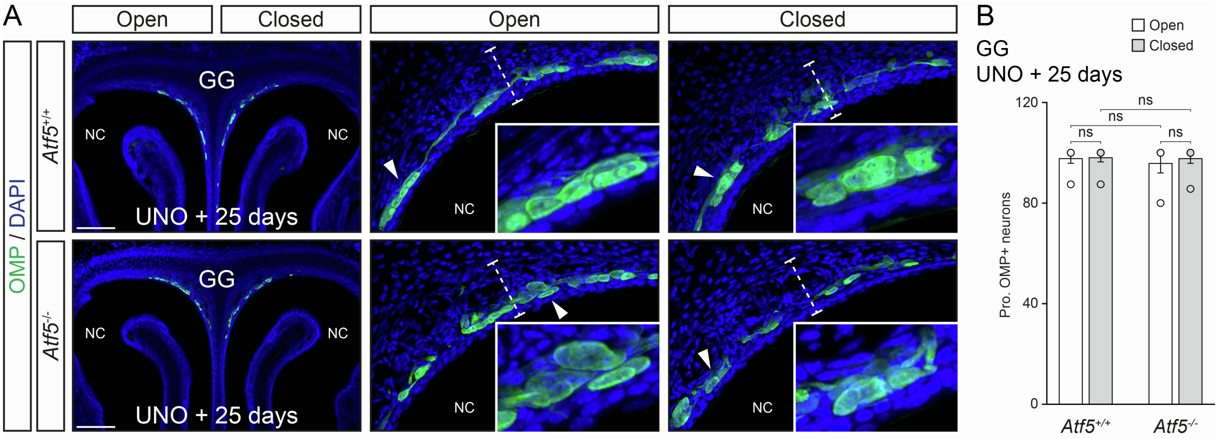

Figure S7. Neuronal maturity of the GG is not affected by the *Atf5* genetic invalidation or by increased environmental stimulations. Related to Figure 6.

(A) Representative immunohistochemistry for the OMP (in green) in the GG of $Atf5^{+/+}$ (in white) and $Atf5^{-/-}$ (in gray) mice after 25 days of UNO. White arrowheads indicate the zoom-in view.

(B) The precise quantifications of the proportion of OMP+ neurons for the GG per genotype and condition (Open and Closed) are shown. No significant difference between genotypes concerning the proportion of OMP+ neurons in the Open side ($Atf5^{+/+}$: N_{mouse} = 3, n_{slice} = 6, 97.9 ± 2.1%; $Atf5^{-/-}$: N_{mouse} = 3, n_{slice} = 5, 96.0 ± 4.0%; ns) as well as in the Closed side ($Atf5^{+/+}$: N_{mouse} = 3, n_{slice} = 7, 98.2 ± 1.8%; $Atf5^{-/-}$: N_{mouse} = 3, n_{slice} = 7, 98.0 ± 2.0%; ns). The GG epithelium width designated with white dashed lines did not significantly change between naris sides and/or genotypes (Open side $Atf5^{+/+}$: N_{mouse} = 3, n_{slice} = 6, 55.5 ± 7.3 μm; Open side $Atf5^{-/-}$: N_{mouse} = 3, n_{slice} = 5, 56.9 ± 2.6 μm; ns | Closed side $Atf5^{+/+}$: N_{mouse} = 3, n_{slice} = 7, 55.3 ± 4.4 μm; Closed side $Atf5^{-/-}$: N_{mouse} = 3, n_{slice} = 7, 56.1 ± 4.2 μm; ns). For the morphological aspect, nasal cavities (NC) are indicated in (A). Nuclei are counterstained with dapi (DAPI, in blue) in (A) and are used to quantify the proportion (Pro.) of OMP+ neurons according to the number of DAPI+ nuclei in (B). Scale bars are 200 μm in (A). Data are expressed as mean ± SEM with aligned dot plots for ≥ 5 slices emerging from at least 3 different mice per genotype in (B). For comparisons between conditions and genotypes, two-tailed Student's *t*-test or Mann-Whitney *U*-test are used, ns for non-significant in (B).

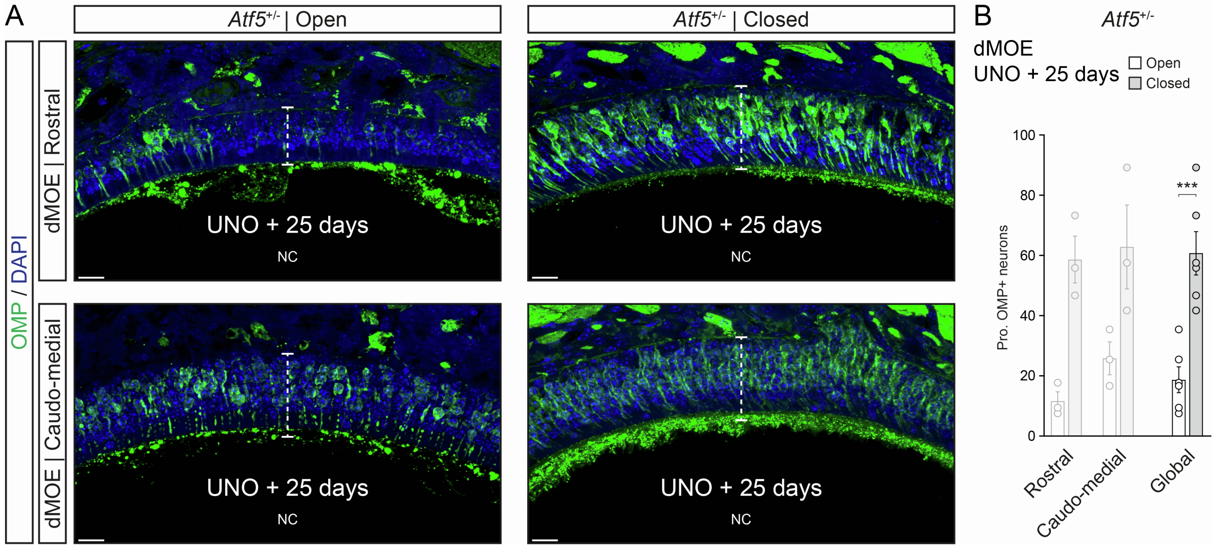

Figure S8. Heterozygous *Atf5*$^{+/-}$ mice display a rostral-caudal axis-dependent MOE response under increased environmental stimulations. Related to Figure 6.

(A) Representative immunohistochemistry performed on cryosections of OMP (green) in the dMOE of an *Atf5*$^{+/-}$ adult mouse (PM5) after 25 days of unilateral naris occlusion (UNO), shown in the rostral and caudo-medial regions.

(B) Quantifications of the proportion of OMP+ neurons along different regions of the MOE rostral-caudal axis, for the Open (white) and Closed (gray) sides. Nasal cavities (NC) are also indicated to highlight the morphological context in (A). Nuclei are counterstained with DAPI (blue) in (A), and were used to determine the proportion (Pro.) of OMP+ neurons relative to total DAPI+ nuclei in (B). Scale bars: 20 μm in (A).

Confocal acquisitions of the cryosections were made with Leica Stellaris 8 in (C) and (D). Data in (B) are presented as mean ± SEM with aligned dot plots representing 3 sections per region (rostral and caudo-medial), from 1 mouse. For statistical analysis, data were also pooled across regions (Global). Comparisons between conditions and genotypes were performed using two-tailed Student's *t*-tests in (B), ***$p<0.001$.

GENE AND ALLELE	FORWARD PRIMER	REVERSE PRIMER
PCR experiments		
Omp$^{+/+}$	5'–GAAGCAGCAGCTGGAGATG–3'	5'–GCATCCGGCTTCTAGACT–3'
Omp$^{-/-}$	5'–CAGCGTGCAGCTCGCCGACC–3'	5'–GCAGCATCCGGCTTCTAGACT–3'
Atf5$^{+/+}$	5'–GTATGATTGCCTGGTTGGCTGGTTA–3'	5'–CACTTCGGTGTTCACTCATGTCCCA–3'
Atf5$^{-/-}$	5'–GTATGATTGCCTGGTTGGCTGGTTA–3'	5'–GTCACCTGTCATGTTCTTGCCAAGTG–3'
RT-PCR experiments		
Atf5	5'–GGCTGGCTCGTAGACTATGG–3'	5'–CCAGAGGAAGGAGAGCTGTG–3'
Gapdh	5'–AACTTTGGCATTGTGGAAGG–3'	5'–ACACATTGGGGGTAGGAACA–3'
RT-qPCR experiments		
Atf5	5'–GGCTGGCTCGTAGACTATGG–3'	5'–CCAGAGGAAGGAGAGCTGTG–3'
Gapdh	5'–AACTTTGGCATTGTGGAAGG–3'	5'–ACACATTGGGGGTAGGAACA–3'

Table S1. Sequences of primers used for PCR, RT-PCR and RT-qPCR experiments. Related to STAR Methods.
